# Autologous haematopoietic stem cell transplantation for rheumatic diseases: best practice recommendations from the EBMT Practice Harmonization and Guidelines Committee

**DOI:** 10.1038/s41409-025-02695-y

**Published:** 2025-08-20

**Authors:** Tobias Alexander, Elisa Roldan, Nicoletta Del Papa, Dominique Farge, Jörg Henes, Zora Marjanovic, Mathieu Puyade, John A. Snowden, Julia Spierings, Jeska K. de Vries-Bouwstra, Francesco Onida, Annalisa Ruggeri, Isabel Sánchez-Ortega, Richard Burt, Ricard Cervera, Andrea Doria, John Moore, Maria Carolina Oliveira, Grégory Pugnet, Doron Rimar, Marc Schmalzing, Ibrahim Yakoub-Agha, Raffaella Greco

**Affiliations:** 1 Department of Rheumatology and Clinical Immunology, Charité - Universitätsmedizin Berlin, corporate member of Freie Universität Berlin, Humboldt-Universität zu Berlin, and Berlin Institute of Health, Berlin, Germany; 2 German Rheumatism Research Centre, a Leibniz Institute, Berlin, Germany; 3 Department of Haematology, Sheffield Teaching Hospitals NHS Foundation Trust, Sheffield, UK; 4 Division of Clinical Medicine, School of Medicine and Population Health, The University of Sheffield, Sheffield, UK; 5 Scleroderma Clinic, Rheumatology Dept, ASST G. Pini-CTO, Università degli Studi di Milano, Milano, Italy; 6 Internal Medicine Unit (04): CRMR MATHEC, Maladies Auto-immunes et Thérapie Cellulaire, Centre de Référence des Maladies auto-immunes systémiques Rares d’Ile-de-France, AP-HP, St-Louis Hospital, INSERM UMR1342, IRSL, Paris-Cite University, Paris, France; 7 Department of Medicine, McGill University, Montreal, QC Canada; 8 University hospital Tuebingen Tübingen, Internal Medicine II (Hematology, Oncology, clinical immunology and rheumatology) Tübingen, Tübingen, Germany; 9 Service d’Hématologie Clinique et Thérapie Cellulaire, Hôpital Saint-Antoine, AP-HP, Paris, Sorbonne University, Paris, France; 10 CHU de Poitiers, Service de Médecine Interne et Maladies Infectieuses, Poitiers, France; 11 Department of Rheumatology and Clinical Immunology, University Medical Center Utrecht, Utrecht, The Netherlands; 12 Department of Rheumatology, Leiden University Medical Center, Leiden, The Netherlands; 13 Hematology & ASCT Unit, ASST Fatebenefratelli-Sacco, University of Milan, Milan, Italy; 14 Hematology and BMT Unit, San Raffaele Scientific Institute, Milano, Italy; 15 Secretary of the Practice Harmonization and Guidelines committee of EBMT, Barcelona, Spain; 16 EBMT Medical Officer, Executive Office, Barcelona, Spain; 17 Northwestern University (retired), Scripps hematology, La Jolla, CA USA; 18 Department of Autoimmune Diseases. Reference Centre for Systemic Autoimmune Diseases, Vasculitis and Autoinflammatory Diseases, Member of ERN-ReCONNET/RITA, Hospital Clínic, University of Barcelona, Barcelona, Catalonia Spain; 19 Division of Rheumatology, Department of Medicine DIMED, University of Padua, Padua, Italy; 20 Department of Haematology and Bone Marrow Transplantation, St Vincent’s Hospital, Sydney, NSW Australia; 21 Center for Cell-based Therapy, Regional Hemotherapy Center of the Ribeirão Preto Medical School, University of São Paulo, Ribeirão Preto, Brazil; 22 Department of Internal Medicine, Ribeirão Preto Medical School, University of São Paulo, Ribeirão Preto, Brazil; 23 Service de Médecine Interne et Immunologie Clinique, CHU Rangueil, Toulouse cedex 9, France; 24 Rheumatology Unit, Bnai Zion Medical Center, Faculty of Medicine, Technion Israel institute of technology, Haifa, Israel; 25 Department of Internal Medicine 2, Rheumatology/Clinical Immunology, University Hospital Würzburg, Würzburg, Germany; 26 CHU de Lille, Univ Lille, INSERM U1286, Infinite, Lille, France; 27 Unit of Hematology and Bone Marrow Transplantation, IRCCS San Raffaele Scientific Institute, Vita-Salute San Raffaele University, Milan, Italy

**Keywords:** Risk factors, Stem-cell research

## Abstract

Immune-mediated rheumatologic and musculoskeletal diseases (RMDs) comprise a heterogeneous group of systemic conditions that affect the connective tissues of the musculoskeletal system and internal organs. Immune-mediated RMDs are driven by chronic autoimmune responses and typically require continuous or repeated administration of immunosuppressive or biologic disease-modifying drugs. Although generally effective, these therapies can cause both short- and long-term side effects and may fail to control the disease with risk of irreversible tissue damage. For such patients, haematopoietic stem cell transplantation (HSCT) has been successfully employed over the past 30 years, but this procedure requires caution due to significant side effects. To address these aspects, updated recommendations for the use of HSCT in RMDs have been developed in collaboration with an international expert panel from the European Society for Blood and Marrow Transplantation (EBMT). The panel reviewed all available evidence regarding HSCT application since 2004. Based on this review, EBMT expert-based consensus recommendations were formulated to guide best practices and ensure high-quality patient care. These recommendations include detailed indications, contraindications, and cautionary notes specific to each RMD, along with comprehensive protocols for diagnostic work-up. They are intended to support clinicians, scientists, patients, and caregivers in the field of RMDs.

## Introduction and current state-of-the-art

Immune-mediated rheumatologic and musculoskeletal diseases (RMD) may affect joints, muscles, internal organs, skin, vessels or connective tissues. These diseases may result in pain, loss of function and reduced quality-of-life and are among the most disabling and costly conditions in the adult population in the developed world [1–3]. RMDs result from a break of immunologic self-tolerance leading to activated autoreactive T and B cells with consequent development of autoantibodies. Most of the classic RMDs are polygenic and exhibit features of the broad spectrum of autoimmune and autoinflammatory mechanisms [4, 5]. Examples for severe forms of autoimmune RMDs are systemic diseases such as systemic sclerosis (SSc) [6], systemic lupus erythematosus (SLE) [7], idiopathic inflammatory myopathies (IIM) [8], vasculitis and rheumatoid arthritis (RA). The use of immunosuppressive, biologic, or targeted synthetic disease-modifying drugs (DMARDs), administered as monotherapies or in combination, is recommended by the European Alliance of Associations for Rheumatology (EULAR) guidelines for treatment, depending on the individual manifestations and severity of the disease [9–12].

However, not all RMD patients sufficiently respond to conventional or biologic therapies, which can lead to disease flares, progression, and ultimately morbidity and mortality. In this context, restoration of immunologic tolerance with consequent resolution of autoimmune and inflammatory responses against self-antigens is key to provide durable remissions in RMD patients [13]. Over the last four decades, there has been increasing clinical and scientific evidence that immunological tolerance can be achieved by the use of high-dose cytotoxic therapy combined with serotherapy, followed by autologous, or less frequently allogeneic, hematopoietic stem cell transplantation (HSCT). This approach allows for resetting the immune response and re-inducing immunologic self-tolerance, a concept developed and utilized over nearly four decades, beginning with animal models [14, 15]. The clinical application of HSCT has become an integral part of standard-of-care treatment algorithms for certain indications, particularly for patients with rapidly progressive, early severe SSc [9, 16]. Previous European Society for Blood and Marrow Transplantation (EBMT) guidelines and recommendations have recommended that all patients being considered for and treated with HSCT are managed in institutions where relevant autoimmune disease specialists work closely together with transplant hematologists and others in a multidisciplinary team (MDT), in accordance with JACIE (or equivalent) accreditation standards and other regulatory requirements [17].

Given the continually evolving evidence base in the field, with numerous novel treatment options, there is a need for updated EBMT clinical practice guidelines and recommendations to support both the RMD and haematopoietic cell transplantation (HCT) communities and their patients at various levels, including national and international organizations, as well as local clinical teams across rheumatologic specialties who may refer patients. This EBMT consensus aims to promote patient safety and harmonize procedures for AD patient selection, care, follow-up, clinical and immune monitoring before and after treatment delivery, and data collection, in accordance with Good Clinical Practice (GCP), Good Manufacturing Practice (GMP), where applicable, and relevant accreditation and regulatory requirements. As with the previous clinical development of cellular therapies for RMDs [18], the EBMT and the broader autoimmune disease specialist community should continue to play a central role in coordinating retrospective registry-based analyses and prospective studies to evaluate the safety and efficacy of HSCT in patients with RMDs.

## Methods

### Methodology

These consensus recommendations were developed by an international panel of experts during a 2-day in person workshop held in Lille, France, in September 2024. This workshop was conducted following the methodology published by the EBMT Practice Harmonization and Guidelines Committee, with the aim of providing clear and practical recommendations based on international consensus or, where such consensus is lacking, expert opinion [19]. Nineteen experts from different countries belonging to the EBMT, including the Autoimmune Diseases Working Party (ADWP), and international experts were invited to join the workshop. Two videoconferences were held in preparation of the workshop, during which specific research questions and areas to be covered were developed and confirmed. Previous ADWP guidelines [17, 20–22], ADWP best practice recommendations for management of innovative cellular therapies [18], and other international guidelines [23, 24] provided a model for discussions. After the in-person meeting in Lille, a draft paper was generated and further circulated to the experts for review and contribution. They therefore represent the consensus views of all the authors. Levels of evidence have been inserted according to the currently accepted EBMT grading system (Table 1) [16].

**Table 1:** EBMT categorisation of type of indication for transplant procedures and strength of evidence [16].

Categories	Settings where HSCT ought to be performed
Standard of care (S)	Indications reasonably well defined and results compare favourably (or are superior) to those of non-transplant treatment approaches. Obviously, defining an indication as the standard of care does not mean an HCT is necessarily the optimal therapy for a given patient in all clinical circumstances. ‘Standard of care’ transplants may be performed in a specialist centre with experience in HCT and an appropriate infrastructure as defined by the JACIE standards.
Clinical option (CO)	Indications for which the results of small patient cohorts show efficacy and acceptable toxicity of the HCT procedure, but confirmatory randomised studies are missing, often as a result of low patient numbers. The broad range of available transplant techniques combined with the variation of patient factors such as age and co-morbidity makes interpretation of these data difficult. Our current interpretation of existing data for indications placed in this category supports that HCT is a valuable option for individual patients after careful discussions of risks and benefits with the patient, but that for groups of patients, the value of HCT needs further evaluation. Transplants for indications under this heading should be performed in a specialist centre with major experience in HCT with an appropriate infrastructure as defined by JACIE standards.
Developmental (D)	Indications when the experience is limited, and additional research is needed to define the role of HCT. These transplants should be done within the framework of a clinical protocol, normally undertaken by transplant units with acknowledged expertise in the management of that particular disease or that type of HCT. Protocols for D transplants will have been approved by local research ethics committees and must comply with current international standards. Rare indications where formal clinical trials are not possible should be performed within the framework of a structured registry analysis, ideally an EBMT non-interventional/observational study. Centres performing transplants under this category should meet JACIE standards.
Generally not recommended (GNR)	Comprises a variety of clinical scenarios in which the use of HCT cannot be recommended to provide a clinical benefit to the patient, including early disease stages when results of conventional treatment do not normally justify the additional risk of an HCT, very advanced forms of a disease in which the chance of success is so small that does not justify the risks for patient and donor, and indications in which the transplant modality may not be adequate for the characteristics of the disease. A categorisation as GNR does not exclude that centres with particular expertise on a certain disease can investigate HCT in these situations. Therefore, there is some overlap between GNR and D categories, and further research might be warranted within prospective clinical studies for some of these indications.
**Grade**	**Strength of the evidence supporting the assignment of a particular category**
Grade I	Evidence from at least one well-executed randomised trial.
Grade II	Evidence from at least one well-designed clinical trial without randomisation; cohort or case-controlled analytic studies (preferably from more than one centre); multiple time-series studies; or dramatic results from uncontrolled experiments.
Grade III	Evidence from opinions of respected authorities based on clinical experience, descriptive studies, or reports from expert committees.

### Objectives and research questions

These recommendations were developed due to the growing number of autologous HSCT performed for RMDs [14]. They aim to cover detailed indications, contraindications, and cautionary notes specific to each RMD, along with comprehensive protocols for diagnostic work-up, clinical management, and immune monitoring during HSCT. The recommendations focussed on the following research questions: (i) Which RMD patients are candidates for autologous HSCT? (ii) What is the optimal timing for auto-HSCT in RMD patients? (iii) How do we perform autologous-HSCT in RMD patients? (iv) How do we manage RMD patients post-autologous HSCT? They represent the consensus point of view of expert authors from international MDTs. They reflect current best practices in this growing field and aim to help clinicians and other healthcare professionals in providing consistent, high-quality patient care and support local clinical teams delivering these specialized treatments. These EBMT recommendations are intended to be general in scope and applicable to all mentioned RMDs and autologous HSCT adopted within current clinical practice. These guidelines and recommendations should not inhibit progress and innovation but instead used as a basis for further research and development of conditioning regimens, standards of care, inclusion or exclusion of patient groups, biomarkers, heterogeneity in disease, end organ regeneration, or inclusion of artificial intelligence. When administering HSCT for RMD within clinical trials, physicians are advised to follow respective trial protocols.

### Search strategy and selection criteria

Data for literature review were identified by searches of MEDLINE, Current Contents, PubMed, and references from relevant articles using the search terms “autologous HSCT”, “rheumatological diseases” and “systemic sclerosis” or “SLE”, lupus”, “myositis”, “vasculitis” or “arthritis” (Supplementary material). Only articles written in English from January 2004 until September 2024, including all clinical (single or randomized phase II, or phase III randomized controlled) trials as well as registry analyses and key reviews/guidelines were considered in the evaluation, and served as the basis for the discussions. In rare disease indications, given the lack of high-quality evidence from randomized trials, we also included case series and older data supporting the use of HSCT.

## Results

### General considerations

#### General recommendations

Evidence for the feasibility, efficacy and toxicity of HSCT in RMDs has been provided by many clinical trials, large registry analyses and summarized in previous reviews [14, 25, 26]. The risks of toxicity and transplant-related mortality (TRM) may vary between autologous and allogeneic haematopoietic stem cell (HSC), intensity of conditioning regimens, centre experience, patient selection, and RMD category [25, 27, 28]. The potential for safer, yet equally effective, non-HSCT treatments should be actively explored in all cases, including biological therapies, where deliverable. Disease activity, organ damage and organ involvement should be carefully assessed before HSCT in RMDs. All clinical and paraclinical parameters necessary to evaluate all comorbidities associated with the systemic AD activity, damage and severity should be documented, with a detailed work-up dating back to no more than three months before the planned HSCT procedure. Specific indications are given for each RMD in subsequent sections. Clinicians should consider the balance between active disease and the possibility of withdrawing immunosuppressive therapies within the time window required to perform HSCT, as well as the importance of sequelae secondary to RMD. Non-interventional prospective studies offer a means of obtaining meaningful clinical data, where full phase III randomized controlled trials are not feasible, and are the preferred option over ‘ad-hoc’ procedures (level III).

**Bullet points:**

- A MDT, with at least one disease and transplant specialist is mandatory to discuss eligibility and follow the patient during and after the transplant procedure (level III).
- In addition to JACIE certification (or equivalent), centres should specifically train staff (physicians, nurses, allied health staff, data managers) in the management of specific RMDs, since it is linked to improved outcomes (level III).
- Whenever possible, patients considered for HSCT should be entered into a Research Ethics Committee/Institutional Review Board (REC/IRB) approved clinical trial or prospective non-interventional study (level III).
- All autologous and allogeneic HSCT cases should be reported to international registries (e.g. EBMT or other equivalent societies) (level III).
- Age above 65 years should not be a specific limitation for HSCT treatment per se, but co-morbidities may increase with age and should be considered as part of the biological fitness of the patient for HSCT (level II).
- Impact of the conditioning regimen on short- and long-term outcomes is an important consideration in the planning of HSCT in ADs (level III).
- Careful and standardized follow-up procedures (for the first 100 days and at least 6 monthly for the first two years, and afterwards annually if medically stable) after HSCT are important to assess and monitor the AD patient, disease evolution and the immunological reconstitution process. Other healthcare structures, including follow-up and rehabilitation care centres, may be involved with other healthcare service providers (i.e. nutrition and medical equipment) (level III).
- Long-term formalized follow-up is a minimum recommendation. Annual review and data reporting are recommended to capture all outcomes, including late effects of HSCT (level III).
- Biobanking within current regulatory frameworks allowing to maximize the utility of stored biological samples should be actively sought (level II).
- Studies using other sources of data (registry and established clinical trials) should be used in evaluating the potential cost-effectiveness of HSCT compared with non-HSCT treatment options (level II).

#### General eligibility criteria

When using HSCT in patients with RMD, an acceptable balance between the expected benefits and potential side effects should be considered, based on available evidence and considering alternative treatment options. Therefore, potential candidates for HSCT should generally have failed one or more lines of standard (guideline-based and/or regulatory-approved) therapies, a severe rapidly progressive disease with poor prognosis (according to disease specific risk scores for disease related mortality), and a general condition that permits HSCT with minimal risk of morbidity and mortality. Under these considerations, general considerations (Table 2) and contraindications (Table 3) have been developed.

**Table 2:** General considerations for HSCT in RMDs.

Criterion	Specific recommendations for ADs
Age	18–65 years old. In exceptional cases, older patients could be considered along with co-morbidity assessment and the agreement of the MDT (level III).
Disease status and evaluation	HSCT should be considered as a therapeutic option in patients with RMDs, which are active or progressive despite the use of standard (guideline-based and/or regulatory approved) therapy (level II).
Centre and team requirement	In patients for whom HSCT represents a treatment option, referral should be made to a centre with an appropriate experience combining haematological and RMD specialist teams. These units should be experienced in selecting and managing severe and refractory RMD patients. Such centres should have established multidisciplinary team meetings and/or similar processes for HSCT, involving both a RMD and haematology focused program in the same centre to support thorough assessment, treatment and follow-up (level II).
General clinical assessment	Performance status, HCT-CI [86], QoL questionnaires [87], smoking absence (with date of cessation if applicable), weight/height/body mass index, blood pressure/heart rate, full physical examination and nutritional assessment should be assessed (level III). Careful MDT evaluation is needed for patients with poor performance status (ECOG ≥2, Karnofsky ≤60%) related to the underlying disease likely to reverse after HSCT (level III).
Patient compliance	Patient compliance and understanding of the procedure and expectations is essential, as a basis for providing informed written consent for HSCT procedure and sharing registry data (level III). Social service and psychological assessment includes an evaluation for post-traumatic stress disorder and psychosocial support and structure needed to cope with disease treatment and follow up (level III).
Fertility	Fertility assessment and gamete preservation must be proposed to RMD patients before HSCT. Consideration should be given to chemotherapy induced infertility, risk of premature menopause, and ultimate need for hormone replacement therapy, where appropriate. Fertility assessment is mandatory (level III).
Exclusion of pregnancy	Pregnancy should be excluded within 7 days of administering mobilization or conditioning chemotherapy (level III).
Neoplasia screening	A complete assessment to exclude neoplasia should be done according to national recommendations and/or local practices (level III).
Baseline laboratory and imaging assessments	Haematological assessment: complete blood counts, reticulocytes; coagulation; transfusion work-up (including blood group). Biochemistry: liver function, renal function and electrolytes, bone profile, blood protein electrophoresis and albumin, blood glucose, haemoglobin A1c, vitamins, iron status and other relevant nutrients, CRP or other inflammatory parameters. Cardiac assessment: Cardiac biomarkers (Troponin hs, NT-proBNP); ECG plus a 24-h Holter according to the AD type and the presence of any clinical or electrical abnormal signs; transthoracic echocardiography with LVEF, systolic PAP and tricuspid flow measures. Extended cardiac assessment as per SSc section and other disease specific sections should be performed. Lung assessment: CT scan, pulmonary function tests with measurement of haemoglobin-corrected DLCO. Infectious assessment: Search for and/or treatment of any ongoing or past infections as clinically appropriate. Dental assessment less than 4 months. Syphilis and tuberculosis screening. HIV1 and 2, HTLV 1 and 2, HBV, HCV, CMV, HSV, EBV, VZV, toxoplasma serologies. EBV blood PCR and CMV blood PCR. SARS-COV-2 test according to local practice; aspergillosis assessment; further assessments including parasitic according to patient’s travel history to endemic areas should be considered.

*CMV* cytomegalovirus, *CT* computed tomography, *DLCO* diffusing capacity for carbon monoxide, *EBV* Epstein-Barr-Virus, *ECG* electrocardiogram, *ECOG* Eastern Cooperative Oncology Group, *GFR* glomerular filtration rate, *HBV* Hepatitis B virus, *HCT-CI* hematopoietic cell transplantation-comorbidity index, *HIV* human immunodeficiency virus, *HSV* herpes simplex virus, *HTLV* human T-lymphotropic virus, *LVEF* left ventricular ejection fraction, *MDT* multidisciplinary team, *NT-proBNP* N terminal brain natriuretic peptide, *hs* high sensitivity, *PCR* polymerase chain reaction, *QoL* quality of life, *PAP* pulmonary artery pressure, *VZV* varicella zoster virus.

**Table 3:** General contraindications for HSCT in RMDs.

Criterion	Contraindications
Active infections	Active infections and uncontrolled chronic infections are absolute contraindications. Careful consideration should be given for controlled chronic infections.
Malignancy	Active solid neoplasia and haematological malignancies, or cancer diagnosed within the last 5 years, except for cervical ’in situ’ neoplasia (CIN) and non-melanoma skin cancer. For patients in remission for <5 years, HSCT should be discussed individually with the referring oncologist.
Cardiac function	Congestive heart disease with LVEF ≤40% as measured by TTE, uncontrolled arrhythmias, severe non controlled ischemic cardiac disease, pericardial effusion with hemodynamic alterations. For SSc patients, specific contraindications should be considered (see section below).
Lung function	DLCO-SB ≤40%. In some exceptional cases, lower DLCO may be accepted.
Liver and kidney function	Caution is required in patients with increased liver enzymes above 3 ULN and GFR of ≤30 ml/min but alterations in the value should be discussed individually in MDT.
Cytopenia	Defined as: Neutropenia <0.5 × 10^9^/L and/or thrombocytopenia <50 × 10^9^/L and/or CD4^+^ lymphopenia <200 × 10^9^/L. Patients with immune cytopenia associated with the RMD should be considered on an individual basis following bone marrow examination and other relevant investigations to exclude bone marrow disease (i.e. myelodysplastic syndrome, aplastic anaemia). Individual considerations by MDT are recommended. Ethnicity is also a consideration, and neutropenia related to ethnicity is not a contraindication, although full work up including Duffy typing is recommended [88].
Bleeding disorder	Uncontrolled bleeding disorder
Psychiatric disease	Uncontrolled psychiatric disease
Pregnancy	Pregnancy and lack of appropriate contraception during the procedure
Smoking	Active smokers

*DLCO-SB* single-breath diffusing capacity for carbon monoxide, *GFR* glomerular filtration rate, *LVEF* left ventricular ejection fraction, *MDT* multidisciplinary team, *ULN* upper limit of normal, *TTE* transthoracic echocardiography.

#### Recommendations on wash-out period before mobilization specifically for RMDs

Washout of DMARDs needs to be considered due to potential impact on mobilization or increased risk of infections. To this end, a discontinuation of the following drugs is recommended at 2–3 weeks before mobilization: mycophenolate mofetil, azathioprine, calcineurin inhibitors, mTOR inhibitors, JAK-inhibitors, cyclophosphamide, methotrexate and subcutaneous tocilizumab and one week before mobilization: CD20-targeting therapies [18]. It is recommended to perform HSCT within 2 weeks and up to 3 months after apheresis, during which time the HSCT work-up does not need to be repeated and ideally wash out of the immunosuppressive treatment should be maintained, provided there is no change in clinical status. The general exceptions are serological testing (e.g., HIV and hepatitis) and pregnancy tests, which should be updated within seven days prior to prior to the initiation of the recipient’s preparative regimen in accordance with FACT-JACIE standards.

#### Mobilization, stem cell collection and CD34-selection

Over the past decades, mobilized peripheral blood stem cells (PBSCs) have largely replaced bone marrow as the primary source of HSCs for autologous HSCT, because PBSCs contain a significantly higher number of CD34^+^ cells and also offer a more convenient collection procedure and faster hematologic recovery [29]. However, obtaining a sufficient number of autologous PBSCs depends on the effective mobilization of HSCs from the bone marrow niche into the bloodstream, usually using G-CSF and cyclophosphamide. Purification of HSCs from the stem cell grafts using CD34 selection may be performed to reduce the risk of reinfusing autoreactive lymphocytes into the patient, but this purging is associated with excess infection due to delayed immune reconstitution and the selection procedure adds significantly to the costs and logistics of the procedure [30]. While some post-hoc analyses from clinical studies in SLE and SSc suggested improved outcomes in CD34-selected HSCT transplant recipients [30, 31], others have not shown benefit or are inconclusive [32]. In addition, one pilot randomized study of HSCT in rheumatoid arthritis did not demonstrate superiority of CD34 selection [33]. The intensity of the conditioning regimen used and the use of anti-thymocyte globulin (ATG) have been variable across clinical practice, providing challenges for assessment of the utility of ATG. Based on the current level of evidence and understanding, the following recommendations were developed:

- Autologous HSC may be derived from peripheral blood or bone marrow. Mobilized PBSC are preferred based on ease of procurement and better engraftment characteristics (level II).
- Mobilization procedures and stem cell processing should be performed in JACIE or FACT (or equivalent) accredited collection centres (level III).
- Priming chemotherapy is recommended to enhance mobilization, whilst maintaining disease control and to prevent potential flare, which may be a consequence of G-CSF alone (level I). The most commonly used dosage for mobilization is cyclophosphamide at 2 g/m^2^ with uromixetan (Mesna) and cautious hyperhydration followed by G-CSF 5–10 µg/kg/day (level II).
- A minimum dose of 2 × 10^6^/kg CD34^+^ cells should be reinfused, irrespective of any graft manipulation (level II). A higher yield of 2.5–5 × 10^6^ CD34^+^ cells/kg have been associated with faster neutrophil and platelet recovery. The target dose of HSC to be collected should be a minimum of 5 × 10^6^ CD34^+^ cells/kg in case of graft manipulations (e.g. CD34-selection) and a minimum of 2 × 10^6^ CD34^+^ cells/kg in the absence of graft manipulation.
- Back-up harvest can be considered, especially when graft manipulation has been undertaken (level II).
- When cyclophosphamide primed mobilization exceptionally fails, a second attempt at PBSC mobilization or bone marrow harvest should be considered. Despite the lack of evidence in patients with RMD, the use of plerixafor and G-CSF may be reasonable in poor mobilizers after weighing up the benefits and risks, and glucocorticoids may be considered to prevent disease flare (level II).
- There is no consistent evidence from prospective or randomized trials or registry data to support superiority of CD34^+^ or other ex-vivo graft manipulation, although decisions can be made on an individual patient basis (level II). Further research is warranted to determine the benefits, risks and economic aspects of CD34^+^ or other selection based on specific conditioning regimens.

#### Conditioning regimen

Over the past decade, EBMT centres have adopted various conditioning regimens for RMDs (Supplementary material). Three randomized trials comparing conventional therapy of SSc (cyclophosphamide) to autologous HSCT (ASSIST, ASTIS, SCOT) [34–36] demonstrated the superiority of HSCT. An accurate comparison of the different conditioning regimens used in these prospective randomized trials is not feasible due to differences between populations, endpoints [37] and HSCT techniques and would require a randomized trial of the different conditioning regimens. The following recommendations have been developed considering all the relevant literature, including clinical trials [34–36, 38–43], registry studies [27, 44], review/metanalysis [45–47], recommendations/guidelines [17, 48], book chapters [49], and the expertise of the panellists included in the workshop:

- The most commonly used regimen for rheumatologic indications is cyclophosphamide 200 mg/kg (cyclophosphamide 50 mg/kg/day on days –5, –4, –3, –2) in combination with rabbit ATG. Standard conditioning is recommended in presence of: normal cardiac function as assessed by EBMT guidelines [21], normal electrocardiogram (ECG), normal 24 h Holter-ECG, LVEF > 45% at cardiography, absence of right pulmonary hypertension, and no septal bounce on cardiac MRI (level II).
- Reduced dose of cyclophosphamide or combinations with fludarabine plus rituximab (ie. cyclophosphamide 60 mg/kg on day –2, fludarabine 30 mg/m^2^/day on days –5 –4 –3 –2, rituximab 500 mg on days –6 and +2) or thiotepa (i.e. cyclophosphamide 2 × 50 mg/kg and thiotepa 2 x 5 mg/kg) [38], and various rabbit ATG dosing combinations (6.0–7.5 mg/kg), are increasingly used (Supplementary material) (level II). Similar short-term treatment responses have been demonstrated using these regimens with reduction in TRM, likely due to the reduced cardiotoxicity of the conditioning, shorter duration of neutropenia and less transfusion requirement. So-called ‘cardiac safe’ conditioning [38, 39] can be considered in patients: age >50 years, abnormalities on ECG and/or 24 h Holter-ECG [35] and/or echocardiography (left or right ventricular dysfunction, pulmonary hypertension, auricular dysfunction), abnormal right heart catheterization (RHC) and/or MRI criteria for myocarditis [50] or septal bounce on cardiac MRI (level II). Further research and randomized trials are warranted.
- As part of the conditioning regimen, the use of ATG has varied in type and dose in both clinical trials and registry data.
  - In the EBMT ASTIS clinical trial, Thymoglobulin® (Sanofi) rabbit ATG 7.5 mg/kg (administered in equal amounts over 3 consecutive days) was used.
  - Based on the ASSIST and related ‘cardiac safe’ protocols, a reduced total dosage of Thymoglobulin® rabbit ATG, i.e. 6.0–6.5 mg/kg rabbit ATG (administered as 0.5 mg/kg on day –5, 1.0 or 1.5 mg/kg on day –4, and 1.5 mg/kg/day on days –3, –2, –1) is progressively used (level II).
  - Grafalon® (Neovii) rabbit ATG i.e. 10 mg/kg/day on days –3, –2, –1, total dose 30 mg/kg) has been used in some centres (level III).
  - Horse ATG (hATG, ATGAM®, Pfizer) 10 mg/kg on Days –6, –5, –4 and –3 has been used [51] as an alternative to rabbit ATG in conditioning. It may be a consideration in centres where preference over rabbit ATG can be justified.
  - Further research is necessary to determine the optimal type and dosing of ATG and other serotherapy in HSCT conditioning regimens in RMDs (level II).
- Administration of ATG should be done in a slow rate during at least 12 h (ideally 18 h), accompanied by an intermediate dose of glucocorticoids [45]. Details for the dosage, the duration and the tapering of glucocorticoids may vary according to local practise. Based on a recent EBMT-ADWP survey, majority of centres use methylprednisolone (or equivalent, with or without taper) 1 mg/kg/day (37.8% of centres) [45]. Fever and other reactions should be promptly treated according to centre policy and protocols (level III).
- Alemtuzumab is not recommended as serotherapy within the conditioning regimen as it is associated with increased mortality and increased risk of developing secondary autoimmune diseases after HSCT (level II).

- Total body irradiation (TBI) is not recommended because it increases TRM and the risks for myeloid as well as solid malignancies (level II).
- It is recommended to perform HSCT within 2 weeks and up to 3 months after apheresis (level II).
- The interval between the last dose of cyclophosphamide and infusion of the graft should be at least 48 h (level II).
- Patients receiving high doses of cyclophosphamide should receive uromixetan (Mesna) and cautious intravenous hydration as prophylaxis of hemorrhagic cystitis (level I).
- The minimum number of CD34^+^ cells reinfused should be 2.0 × 10^6^/kg, irrespective of graft manipulation (level II).
- Consideration should be given to the short- and long-term toxicities of the various type of serotherapy, including serum sickness (level II).
- Otherwise, supportive care should be based on institutional HSCT practice and standard operating procedures, including maintenance of venous access via central venous catheter from the start of conditioning as below.

#### Supportive care, prophylaxis and follow-up

MDT management of RMD patients undergoing HSCT is highly recommended, with a minimum of one disease specialist and one HCT specialist per centre working closely together [25]. In-patient accommodation for HSCT period is recommended: all patients should be accommodated in isolation facilities, with appropriate clean air facilities in accordance with JACIE accreditation standards during aplasia/severe neutropenia (level II). The risk of infection depends on the underlying AD and degree of immunosuppression [25], and management should be carefully discussed upfront in a MDT meeting (disease specialist, infection-disease specialist, and HSCT specialists). A follow-up of potential infectious complications is considered mandatory. Sufficiently long anti-infectious prophylaxis should be maintained according to patient individual risk and in line with institutional guidelines. Management of these patients should follow specific indications (Table 4), considering all aspects related to the underlying AD and HSCT procedure. Hematologists should be continued to be involved in monitoring of side effects according to ADWP [17] and EBMT Handbook recommendations [52] for the first 100 days and at least 6 monthly for the first two years, and afterwards annually if medically stable with data collection and reporting in the EBMT registry.

**Table 4:** Management of RMD patients undergoing HSCT.

	Specific recommendations in ADs
RBC/platelet transfusions	As for haematological patients, monitoring of blood counts is mandatory in ADs (e.g. at every visit and as clinically indicated), including long-term follow up. Platelet and erythrocyte transfusions should be administered according to centre policy and protocols. Blood products should be irradiated before HSC mobilisation and collection and for a period of time according to institutional and national practice and SOPs, which include consideration of clinical history and immunosuppressive drugs.
G-CSF	In case of grade 3-4 neutropenia, the use of G-CSF may be carefully considered according to the risk/benefit evaluation. Prophylactic administration can be considered (beginning, for example, days +4 to +5 after HSCT infusion), to reduce the duration of neutropenia, according to centre policy.
Infection prophylaxis	Antibacterial prophylaxis: In patients with a high-risk profile for neutropenia, prophylaxis may be considered once ANC <0.5 × 10^9^/L. As per institutional standards. Warning in case of colonization by multi-drug resistant (MDR) pathogens. Anti-viral prophylaxis: All patients. Start from conditioning until 1-year post-HSCT (level II) AND/OR until CD4^+^ count >0.2 × 10^9^/L. Commonly used are valaciclovir 500 mg bid or aciclovir 800 mg bid. Anti-pneumocystis and anti-toxoplasma: All patients. To start from conditioning until 6 months post-HSCT (level III) AND/OR until CD4^+^ count >0.2 × 10^9^/L. Co-trimoxazole (TMP/SMX) 480 mg once daily or 960 mg three doses each week. In case of co-trimoxazole allergy, pentamidine inhalation (300 mg once every month) are recommended, dapsone 100 mg daily or atovaquone 1500 mg once daily can be considered [89]. All patients positive for anti-toxoplasma antibodies should receive oral co-trimoxazole for 6 months (level II) [90]. Systemic primary anti-fungal prophylaxis: The management of anti-fungal prophylaxis should be discussed within a MDT meeting (disease specialist, infection-disease specialist, and HSCT specialists). Anti-fungal prophylaxis should be considered in severe neutropenia (ANC <0.5 × 10^9^/L) and/or prolonged neutropenia for at least 100 days post-transplant (level II). Anti-fungal prophylaxis should be evaluated depending on the duration of glucocorticoid use.
Vaccine strategy	Patients should follow national recommendations on vaccination. Measurements of specific antibody titres may be helpful to determine the need for vaccination after HSCT. Recently, ADWP has also provided specific COVID-19 vaccine recommendations in patients with ADs [91]. Re-vaccinations can be considered starting from 3 to 6 months after HSCT following centre policy and EBMT recommendations [18, 92]. Live vaccines are generally contraindicated in immunosuppressed patients [93].
WBC, biochemistry panel, AST, ALT, LDH, bilirubin, CRP	Standard follow-up as haematological patients. At every visit and as clinically indicated.
CMV, EBV PCR monitoring	Monitor at least during the first 100 days after HSCT, in consideration of immunosuppression. MDT evaluation recommended [94–97]. CMV antibody positive patients receiving ATG or other serotherapy, or receiving manipulated autografts, are recommended to undergo CMV PCR monitoring for the first 100 days after HSCT (level III). CMV reactivation should be treated according to centre policy. EBV antibody positive patients receiving ATG or other serotherapy, or receiving manipulated autografts, are recommended to undergo EBV PCR screening for the first 100 days after HSCT, with active surveillance for post-transplant lymphoproliferative disease according to local practice (level III). Monitoring for CMV and EBV is important and to be considered also in seronegative patients according to local practise (level III).
Quantitative immunoglobulins	Consider immunoglobulin replacement therapy in case of hypogammaglobulinemia (<4 g/l), due to the risk of recurrent infections, after weighing up the benefits, risks and costs of administration (level III).
Late effects testing appropriate to age	Standard follow-up as haematological patients [98]. Yearly or as clinically indicated. The occurrence of secondary ADs [46, 99], e.g. TSH levels for autoimmune thyroid disease or platelets for ITP [100, 101], and secondary malignancies must be monitored.
Fertility, pregnancy and menopause	Risk of pregnancy without birth control and/or premature menopause and infertility should be considered after HSCT [102, 103].
Follow-up after HSCT and ongoing responsibility for the patient	All patients should remain under the direct routine care of the HSCT specialist for at least 100 days after HSCT, or longer, if necessary, until clinically stable. Thereafter combined care between HSCT specialist and referring organ/AD specialist with joint annual review as a minimum is recommended (level III). A follow-up of potential infectious complications should be considered mandatory.

*AD* autoimmune disease, *ADWP* autoimmune diseases working party, *ANC* absolute neutrophil count, *CMV* cytomegalovirus, *EBV* Epstein-Barr-Virus, *G-CSF* granulocyte colony stimulating factor, *HSC* haematopoietic stem cell, *HSCT* haematopoietic stem cell transplantation, *ITP* immune thrombocytopenia, *MDT* multidisciplinary team, *SOPs* standard operating procedures, *TSH* thyroid stimulating hormone.

#### Immunological monitoring after HSCT for RMDs

Immune monitoring should include a baseline assessment (before mobilization and before conditioning) and at intervals after HSCT such as at 3, 6 and 12 months for the first year, then every 6 months for the second year and then annually up to 5 years [20]. Recommended tests include:

• Flow cytometric immunophenotyping of peripheral blood mononuclear cells to measure changes in CD3^+^, CD4^+^ and CD8^+^ T cells, CD19^+^ B cells, NK cells (CD16^+^ and/or CD56^+^) and monocytes (CD14^+^ and/or CD16^+^)

• Serum immunoglobulin electrophoresis (IgG, IgA and IgM quantitation, including monoclone, if present)

Recommendations for the assessment of disease biomarkers are provided in the specific sections below.

### Disease-specific considerations

#### Systemic sclerosis

Systemic sclerosis (SSc) is a rare and devasting autoimmune disease characterized by tissue fibrosis, an immune mediated endothelial vasculopathy and autoantibody production [6]. Despite recent improvements in the disease management [9], mortality is still high, especially in patients with heart involvement, interstitial lung disease (ILD), renal crisis or high modified Rodnan skin score (mRSS) [53, 54]. Over the past years, rapidly progressive diffuse SSc has become the main indication for HSCT among RMD patients. Three RCTs comparing autologous HSCT to intravenous cyclophosphamide demonstrated significant survival benefit, improvements in skin scores, lung-function, and quality of life [34–36]. The European (ASTIS) [35] and American (SCOT) [36] multicentre trials reported 5-year progression-free survival rates of 70–74%, which remained superior to those achieved with monthly cyclophosphamide in the ASTIS trial over a 10-year follow-up period. Based on this evidence, SSc is recommended as a standard indication for autologous HSCT (level I) by the EULAR since 2009 [55], and further supported by updated international 2020 CIBMTR [23] and 2023 EULAR [9] recommendations.

The higher early toxicity of autologous HSCT in SSc compared to other conditions is primarily linked to right ventricular cardiac dysfunction from SSc-related cardiac involvement, especially in cases with pulmonary arterial hypertension, left ventricular diastolic dysfunction from a non-relaxing stiff myocardium, and pericardial constriction or tamponade. Myocardial alterations are at high risk of exacerbation during HSCT by cyclophosphamide cardiotoxicity, fluid hydration and fever. Improved pre-transplant cardiac screening protocols [21], use of conditioning regimens that minimize cyclophosphamide dose [38, 39], and maintaining baseline fluid balance have reduced TRM from 10% in the early ASTIS study [35] to 6% in a prospective non-interventional EBMT study (NISSC-1) [31], 3% in the SCOT study [36], and 2.4% in the CAST study [39].

Eligibility for HSCT varies by protocol but risk factors for disease related mortality and aggressive diseases have been identified. They include progressive SSc (increasing skin score or declining pulmonary function), age at onset <40 years, male gender, antibodies against topoisomerase I or RNA polymerase III, elevated level of C-reactive protein (>10 mg/l), recurrent digital ulcers, tendon friction rubs, weight loss (>10% during the past three months), active myositis on MRI or elevated creatine kinase levels (>2 x ULN), early (during the first year) lung manifestations with DLCO <60% and/or FVC <70% and cardiac manifestations with elevated troponin levels and/or myocardial involvement [54, 56].

Several conditioning regimens have been used for autologous HSCT in SSc, mostly comprising cyclophosphamide and ATG at different doses with a general trend towards lower-intensity regimens. Expert consensus suggests that regimen need to be adapted to the individual cardiopulmonary function of patients. While cyclophosphamide at doses of 200 mg/kg is considered the standard, reduced doses need to be considered in case of cardiac involvement, e.g. using the protocol from the CAST study [39] or thiotepa-based regimens [38], where available. In highly active and severely affected patients with transient contraindications (e.g. active myocarditis), data support pretreatment with RTX and MMF [57]. The CAST regimen was initially applied for patients excluded from high-dose cyclophosphamide transplant due to cardiac parameters of pulmonary artery systolic pressure (PASP) >45 mmHg or mean pulmonary artery pressure (mPAP) >24 mmHg on right heart catheterization, and/or MRI criteria for active myocarditis [50]. Due to the CAST regimen’s reduced toxicity in high cardiac risk transplants, several centres have extended the CAST regimen to include standard cardiac risk patients (level II) [38, 39].

Other concerns unique to HSCT for SSc are gastric antral vascular ectasia (GAVE), renal crises and a general concern of regimen-related post-transplant lymphoproliferative disease (PTLD). GAVE, often referred to as “watermelon stomach” due to the characteristic red streaking of submucosal telangiectasias, is more commonly observed in patients with anti-RNA polymerase III antibodies at the onset of the disease. Iron deficiency anaemia is a common consequence of GAVE and endoscopy should be performed in such patients to assess for GAVE (or any other upper GI cause) before HSCT, given the bleeding risks of thrombocytopenia. Renal crises may be precipitated by the use of glucocorticoids with ATG or cyclophosphamide. Prophylaxis with an angiotensin-converting enzyme inhibitor can be considered, particularly in patients with anti-RNA polymerase III antibodies. Disease-specific considerations are summarized in Table 5.

**Table 5:** Considerations for HSCT in systemic sclerosis.

Inclusion criteria	Exclusion criteria	Concerns	Specific disease assessments
• Age: Recommended age limit is 60 years [39], certain patients >60 years may be considered along with co-morbidity assessment and the agreement of the MDT (level III) • Patients with diffuse progressive SSc fulfilling the 2013 ACR/EULAR classification criteria [104] • Optimal time-point: disease duration <5 years (level I); in case of severe progressive disease, HSCT can be considered after 5 years of disease duration, according to local experience (level III) • Second-line option for patients with evidence of sustained disease activity after 6 months of first-line therapy or intolerance of treatment (level I) • mRSS >15 and visceral involvement with at least 1 organ manifestation OR mRSS >20 without visceral organ involvement but evidence of inflammatory disease (increased CRP >10 mg/L without evidence for infection or other cause) (level I) • Patients with progressive ILD confirmed by chest CT may be eligible in case of mRSS <15 and limited SSc [39] or even sine scleroderma [105] (level III) • HSCT can be considered as first-line option for patients with high risk profile (see red flags above) and rapid progression (study underway) [106] (level III) • In highly active and severely affected patients with transient contraindications (e.g. active myocarditis), data support pretreatment with RTX and MMF [57]	• General contraindications (Table 2) • Cardiac: absolute contraindications for all protocols are cardiac MRI with septal bounce, constrictive pericarditis or cardiac tamponade • Cardiac: absolute contraindications for standard protocols using high-dose cyclo-phosphamide (200 mg/kg) are PASP >40 mmHg at rest or >45 mmHg with fluid challenge, or mPAP >25 mmHg at rest or >30 mmHg with fluid challenge (RHC) according to EBMT reommendations [21] • Cardiac: absolute contraindications for cardiac-safe protocols like the CAST-regimen [39] are LVEF <40%, mPAP >30 mmHg or PASP >50 mmHg in RHC without fluid challenge. • Pulmonary: absolute contraindications are FVC <45% and DLCO-SB <40% • Renal: active renal crisis or creatinine clearance <30 ml/min (for standard protocols); in fludarabine-based regimens dose adjustment in patients with GFR <80 ml/min or according to centre policy • Active smoking	• Caution is required for patients with pulmonary hypertension • Right heart catheterization after 10 days cessation of endothelin-receptor antagonists or calcium channel blockers with fluid challenge (1000 cc normal saline in 10 min i.v. is recommended for exclusion • Reassessment of cardiac function between mobilization and conditioning is recommended, at least by echo-cardiography and ECG, if more than 3-month interval • Caution is required for patients with GAVE	• Blood monitoring should include cardiac biomarkers (CK, NTpro-BNP, troponin hs) • EBV/CMV PCR (at least every two weeks for the 100 days after HSCT (level III) • Skin-score: mRSS [107] • S-HAQ [108] score • Echocardiography with measurement of PAP, LVEF biannually for 2 years, yearly thereafter • Lung function test including FVC, DLCO biannually for 2 years, yearly thereafter • Chest CT scan in case of ILD: annually for the first three years, to be adapted thereafter according to individual involvement • Serology, ANA/ENA, disease-specific, i.e. anti-topoisomerase or anti-RNA polymerase III antibodies, C3, C4 • Consider nailfold capillaroscopy, joint count, range of motion, cardiac MRI, Holter monitor, endoscopy, and oesophageal manometry

*ACR* American College of Rheumatology, *ANA* antinuclear antibodies, *CK* creatine kinase, *CMV* cytomegalovirus, *DLCO-SB* single-breath diffusing capacity for carbon monoxide, *EBV* Epstein-Barr-Virus, *ENA* extractable nuclear antigen antibodies, *EULAR* European Alliance of Associations for Rheumatology, *FVC* forced vital capacity, *GAVE* gastric antral vascular ectasia, *GFR* glomerular filtration rate, *Hs* high-sensitivity, *HSCT* haematopoietic stem cell transplantation, *ILD* interstitial lung disease, *LVEF* left ventricular ejection fraction, *MMF* mycophenolate mofetil, *mPAP* mean pulmonary artery pressure, *MRI* magnetic resonance imaging, *mRSS* modified Rodnan skin score, *NTpro-BNP* N-terminal pro Brain Natriuretic Peptide, *PAP* pulmonary artery pressure, *PCWP* pulmonary capillary wedge pressure, *RHC* right heart catheterization, *RTX* rituximab, *PASP* pulmonary artery systolic pressure, *S-HAQ* Scleroderma Health Assessment Questionnaire.

#### Systemic lupus erythematosus (SLE)

SLE is a prototypic autoimmune disease with heterogenous clinical manifestations, characterized by the development of antibodies directed at a variety of nuclear antigens causing multi-organ inflammation and damage [7]. Despite recent advances in the SLE treatment with approved biologics, such as belimumab and anifrolumab, mortality is still higher than in the general population, especially in youngest patients [58]. According to current EULAR recommendations, treatment should aim at remission [59] or lupus low-disease activity state (LLDAS) [60] with a daily prednisolone maintenance dosage not exceeding 5 mg [10]. Patients not achieving or not maintaining these validated treat-to-target endpoints have a higher risk for developing disease flares, damage accrual and mortality [61, 62].

Data on HSCT is available from single-centre phase I/II clinical trials and retrospective EBMT analyses [30, 63], together covering more than 300 severely affected SLE patients worldwide. Data from these trials indicate a disease-free survival of ~50–65% at 5 years despite discontinuation of DMARDs and responding patients are usually free of clinical symptoms and may regain seronegativity for antinuclear antibodies (ANA) [41, 49, 64]. Early use of HSCT has also been demonstrated to protect against organ-failure and toxicity-related morbidity and to provide improvement in health-related quality-of-life [43]. As for other RMD, cyclophosphamide/ATG based regimens are most commonly used for conditioning (**Supplementary material**). Current data suggest HSCT in SLE as clinical option in patients with active disease despite immunosuppressive and/or biologic therapies (level II) [14]. Disease-specific considerations are summarized in Table 6.

**Table 6:** Considerations for HSCT in SLE and vasculitis.

Inclusion criteria	Exclusion criteria	Concerns	Specific disease assessments
**Systemic Lupus erythematosus**			
• Diagnosis of SLE according to the 2019 EULAR/ACR classification criteria [109] • With at least one active visceral organ or CNS involvement • Active disease reflected by one BILAG [110] A score (severe) or more than 2 BILAG B scores (moderate disease activity) • Failure of treatment with glucocorticoids and at least 2 of the following treatments for at least 3 months: cyclophosphamide, mycophenolate mofetil or its derivatives, anifrolumab, azathioprine, belimumab, methotrexate, obinutuzumab, rituximab, voclosporin or cyclosporin, tacrolimus	• General contraindications (Table 2) • Life-threatening end-organ damage defined as: - FVC <45% and/or DLCO (corrected for Hb) <30% predicted - Advanced liver cirrhosis (Child C) or Bilirubin ≥3 mg/dL (51 µmol/L) - LVEF <40% cardiac echocardiography - Pulmonary hypertension. mPAP pressure ≥20 mmHg measured by RHC	• Caution is required for patients with GFR <30 ml/min. Adjusted conditioning regimen to be considered. • CNS-involvement. • Fertility issue fundamental in usually young female patients	• SLEDAI-2K [111], BILAG [68] or SLE-DAS [112] disease activity scores • ACR SLICC damage index [113] • Organ-specific: CLASI [114] for skin involvement, CDAI for joints, GFR, UPCR and urinary sediment for renal involvement • Serology: ANA/ENA, dsDNA antibodies, serum C3, C4 levels
**Vasculitis**			
*•* Diagnosis of systemic vasculitis according to Chapel-Hill Consensus Conference criteria with life- or organ-threatening involvement • Failure of treatment with glucocorticoids and at least two lines of standard immunosuppressive or immunomodulatory treatment.	• General contraindications (Table 2) • Life-threatening end-organ damage defined as: - FVC <45% and/or DLCO (corrected for Hb) <40% predicted - LVEF <40% cardiac echocardiography	• Caution is required for patients with GFR <30 ml/min. Adjusted conditioning regimen to be considered • Consider risk of vascular damage such as ischemia or dissection.	• BVAS [115] (activity) and VDI [116] (damage) assessment • Serology: c-ANCA, p-ANCA (for AAV) • GFR, UPCR and urinary sediment in patients with renal involvement • CT scan (e.g. in pulmonary granuloma) • PET scan, duplex sonography or MRI in large vessel vasculitis

*ACR* American College of Rheumatology, *ANA* antinuclear antibodies, *ANCA* anti-neutrophil cytoplasmic antibodies, *AAV* ANCA-associated vasculitis, *BILAG* British isles lupus assessment group, Birmingham Vasculitis Activity Score, *CNS* central nervous system, *CRP* C-reactive protein, *CLASI* cutaneous lupus erythematosus disease area and severity index, *CT* computed tomography, *DAS28* disease activity score 28, *DLCO* diffusing capacity for carbon monoxide, *ENA* extractable nuclear antigen antibodies, *ESR* erythrocyte sedimentation rate, *EULAR* European Alliance of Associations for Rheumatology, *FVC* forced vital capacity, *GFR* glomerular filtration rate, *HLH* hemophagocytic lymphohistiocytosis, *ILD* interstitial lung disease, *IVIG* intravenous immunoglobulin, *mPAP* mean pulmonary artery pressure, *MRI* magnetic resonance imaging, *LVEF* left ventricular ejection fraction, *PET* positron emission tomography, *RHC* right heart catheterization, *RTX* rituximab, *SLEDAI* systemic lupus erythematosus disease activity index, *UPCR* urinary protein creatinine ratio, *VDI* vasculitis damage index, *WBC* white blood cell count.

#### Vasculitis

Vasculitides are a group of rare conditions that are characterized by inflammation of blood vessels that demonstrate a wide range of organ involvement and severity, and may lead to long-term sequelae including vision loss, aneurysms or renal failure. The Chapel Hill Consensus definitions, originally published in 1994 and updated in 2012 [65], categorized vasculitis according to the size of affected vessels into large, medium or small vessel vasculitis. Subsequently, modern practical classification criteria for major systemic vasculitides have been developed and endorsed by ACR/EULAR initiatives, including criteria for granulomatosis with polyangiitis (GPA), microscopic polyangiitis (MPA), eosinophilic granulomatosis with polyangiitis (EGPA), giant cell arteritis (GCA) and Takayasu arteritis (TAK) [66]. Similarly, EULAR recommendation for management of the diseases were developed specifically for ANCA-associated vasculitis (AAV) [11] and large vessel vasculitis [67].

In the era of biologic therapies, refractory cases are rare, but rituximab resistance has been reported in patients with GPA [68] and relapse development in GCA under tocilizumab treatment [69]. HSCT has been used as salvage therapy over the past years, including 29 transplants reported to the EBMT registry (**Supplementary material**). Retrospective multicentre analyses from the registry summarizing the outcomes specifically for TAK [70], Behçet’s disease [71] and AAV [72] demonstrated an overall beneficial response to HSCT, but relapses and TRM have been reported. Therefore, HSCT represents a clinical option (level II) in patients with severe and refractory disease, as outlined in Table 6. The recently described VEXAS syndrome, which is associated with vasculitis, polychondritis and other RMD manifestations, should be considered and excluded with molecular (UBA-1) testing as this would be a potential indication for allogeneic HSCT (rather than autologous HSCT) in carefully selected patients [73].

#### Idiopathic inflammatory myopathy

IIM, also commonly referred to as myositis, are a family of rare conditions that are characterized by chronic inflammation of muscle of unknown cause and often include extramuscular manifestations, including rash, arthritis, ILD and cardiac involvement [8]. One of the earliest and most widely used criteria for classifying the IIM has been the Bohan and Peter criteria, but newer EULAR/ACR criteria exist now [74]. However, these criteria have limitations to categorize patients into IIM subtypes, usually comprising dermatomyositis (DM), overlap myositis, anti-synthetase syndrome (ASyS), immune-mediated necrotizing myopathy (MMNM), and sporadic inclusion body myositis (sIBM). The identification of myositis-specific antibodies, which are present in up to 60% of patients, are more useful to classify IIM into homogenous phenotypic subtypes [8]. Treatment of IIM can be challenging as many patients poorly respond to first-line treatment with glucocorticoids, methotrexate, azathioprine or even second-line therapy with cyclophosphamide, rituximab or JAK-inhibitors [75]. For such refractory patients, HSCT was utilized only sporadically over the past decades, delivering mixed results (**Supplementary material**). The majority of the available literature on HSCT in IIM comes from reports of juvenile DM with impressive findings [76, 77], but these were excluded from the current literature search. Disease-specific considerations for IIM are summarized in Supplementary Table 1, and HSCT can only be considered as a ‘clinical option’ (level III) (Table 7).

**Table 7:** Indications for autologous HSCT in RMDs.

Disease	Indication for autologous HSCT
Systemic sclerosis	S/I
Systemic lupus erythematosus	CO/II
Vasculitis	CO/II
Idiopathic inflammatory myopathies	CO/III
Inflammatory arthritis	CO/III

EBMT categorisation of type of indication for transplant procedures and strength of evidence [16].

*S* standard, *CO* clinical option.

#### Rheumatoid arthritis and other inflammatory arthritis

Modern biologic and targeted-synthetic DMARDs revolutionized the treatment landscape in rheumatoid arthritis (RA) [12], and severely refractory patients, usually referred to as difficult-to-treat [78], are the exception. Early in its evolution, HSCT was investigated in severe forms of RA, predominantly in the pre-biologic era. Data from the last retrospective analysis from the EBMT and the Autologous Blood and Marrow Transplant Registry including 73 patients treated between 1996 and 2000 using a cyclophosphamide-based regimen demonstrated an ACR-50 response in only 67% of patients [79]. Treatment with JAK inhibitors achieves almost similar response rates with less toxicity, not justifying continued use of HSCT in RA with primary joint manifestations. Exceptions could be systemic forms of rheumatoid arthritis, including Felty syndrome or Adult-onset Still’s disease (AOSD) with life- or organ-threatening involvement, where positive results have been obtained from individual cases [80]. However, most of these reports derived from juvenile arthritis published before interleukin-1 or interleukin-6 receptor antagonists became available. Therefore, eligibility for HSCT should carefully weigh the risks and benefits according to criteria summarized in Supplementary Table 1, where HSCT could represent a ‘clinical option’ (level III) (Table 7).

## Conclusion and future directions

For over three decades, autologous HSCT has been successfully delivered to RMD patients with severe and treatment-refractory disease courses with more than 3000 cases reported to the EBMT registry [14]. Over time, outcomes have gradually improved due to better patient selection, improved centre experience and optimized supportive care [25]. With accumulating data, the current EBMT recommendations aim to further improve clinical practice by facilitating harmonized procedures for patient selection, transplant management and follow-up. These recommendations reflect currently available evidence, coupled with expert opinion, and will continue to be revised according to necessary modifications in practice. We recommend that decision making is delivered within a MDT, in accordance with GCP and GMP, and appropriate accreditation and regulatory requirements, including JACIE or equivalent accreditation of centres. Data reporting to EBMT and/or equivalent HCT registries is recommended. Where possible, patients should be included into prospective non-interventional studies of the EBMT to evaluate the safety and efficacy in addition to the retrospective registry studies that EBMT regularly performs on available data within the Registry.

The main indication for HSCT in RMDs remains systemic sclerosis, where evidence from RCTs demonstrate autologous HSCT as a ‘standard of care’ (S) indication. Available evidence supports the use of HSCT early in the disease course, especially in those patients with red flags for high SSc-related mortality. Reduced-intensity regimens provided superior safety outcomes and should be considered after thorough pre-transplant work-up with focus on cardiac dysfunction. SLE, vasculitis, IIM and inflammatory arthritis represent indications with clinical option based on evidence from phase I/II trials or smaller cohort studies. Due to the dynamic nature of alternative, highly effective therapies, indications and eligibility criteria for HSCT may change rapidly. Promising results from chimeric antigen receptor (CAR)- T cell studies [81, 82] and first experiences with the utilization of bispecific antibodies [83–85] in major rheumatic diseases have been reported. However, further research is needed to integrate these therapies in current and future treatment algorithms supported by the evidence base. In parallel, scientific studies will be crucial in elucidating the mechanisms of action in HSCT versus CAR-T versus non-cellular therapies in relation to response to treatment, particularly regarding the degree of depletion of autoreactive lymphocytes in inflamed tissues, the reduction of autoantibodies, and the consideration of short-term toxicities alongside long-term risks.

## Supplementary information

Supplementary Document

## Data Availability

The final analysis dataset will be available upon specific request to the Working Party chair.
