# Supplementary material for: Autologous haematopoietic stem cell transplantation for rheumatic diseases: best practice recommendations from the EBMT Practice Harmonization and Guidelines Committee
Source: Bone Marrow Transplant. 2025 Aug 20;60(11):1451–64. doi: 10.1038/s41409-025-02695-y (PMC12583147; doi:10.1038/s41409-025-02695-y)
Supplement: Supplementary file 1 — Supplementary Document [file 41409_2025_2695_MOESM1_ESM.docx]

**Supplementary material to:**

**Autologous haematopoietic stem cell transplantation for rheumatic diseases: best practice recommendations from the EBMT Practice Harmonization and Guidelines committee.**

Tobias Alexander, Elisa Roldan, Dominique Farge, Jörg Henes, Zora Marjanovic, Nicoletta Del Papa, Mathieu Puyade, John A Snowden, Julia Spiering, Jesca K de Vries-Bouwstra, Francesco Onida, Annalisa Ruggeri, Isabel Sánchez-Ortega, Richard Burt, Ricard Cervera, Andrea Doria, John Moore, Maria Carolina Oliveira, Gregory Pugnet, Doron Rimar, Marc Schmalzing, Ibrahim Yakoub-Agha, Raffaella Greco


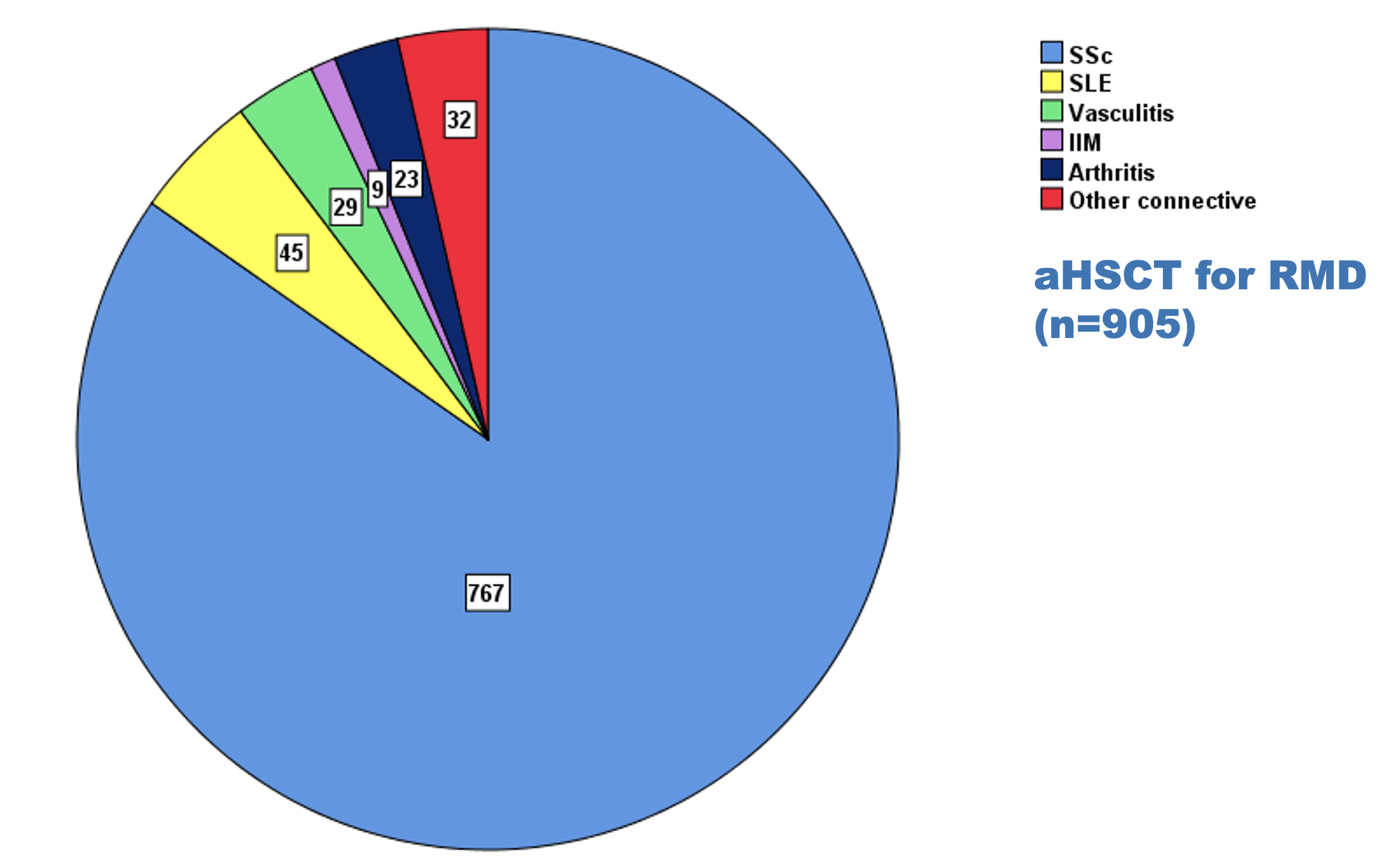


**Supplementary Figure 1:** Number of autologous HCT reported to the EBMT registry for rheumatic diseases in the time between 2004 and July 2023. IIM, Idiopathic inflammatory myopathy; SSc, systemic sclerosis; SLE; systemic lupus erythematosus.

**
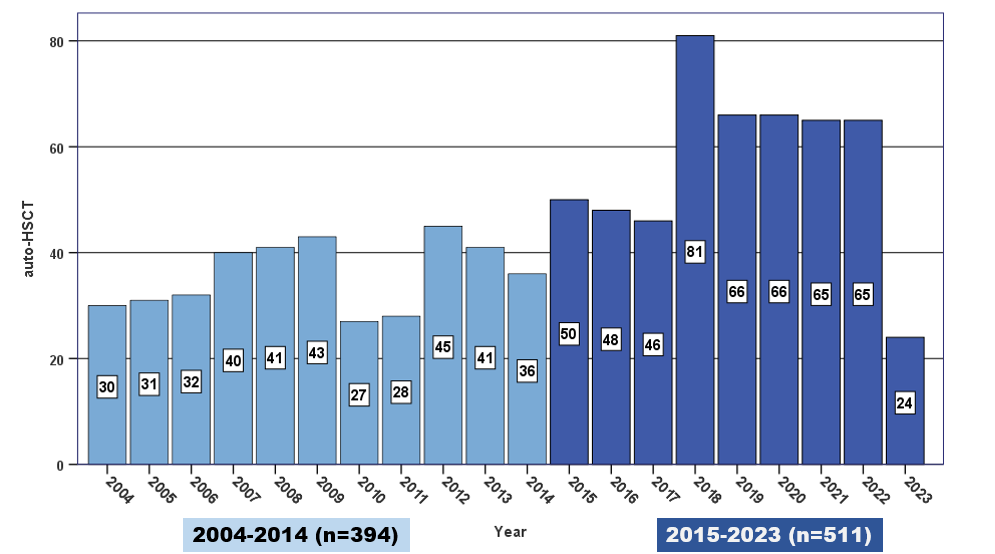
**

**Supplementary Figure 2:**  Transplant activity in the years 2004-2014 vs. 2015-07/2023 based on yearly reported autologous HCT for RMDs to the EBMT registry.


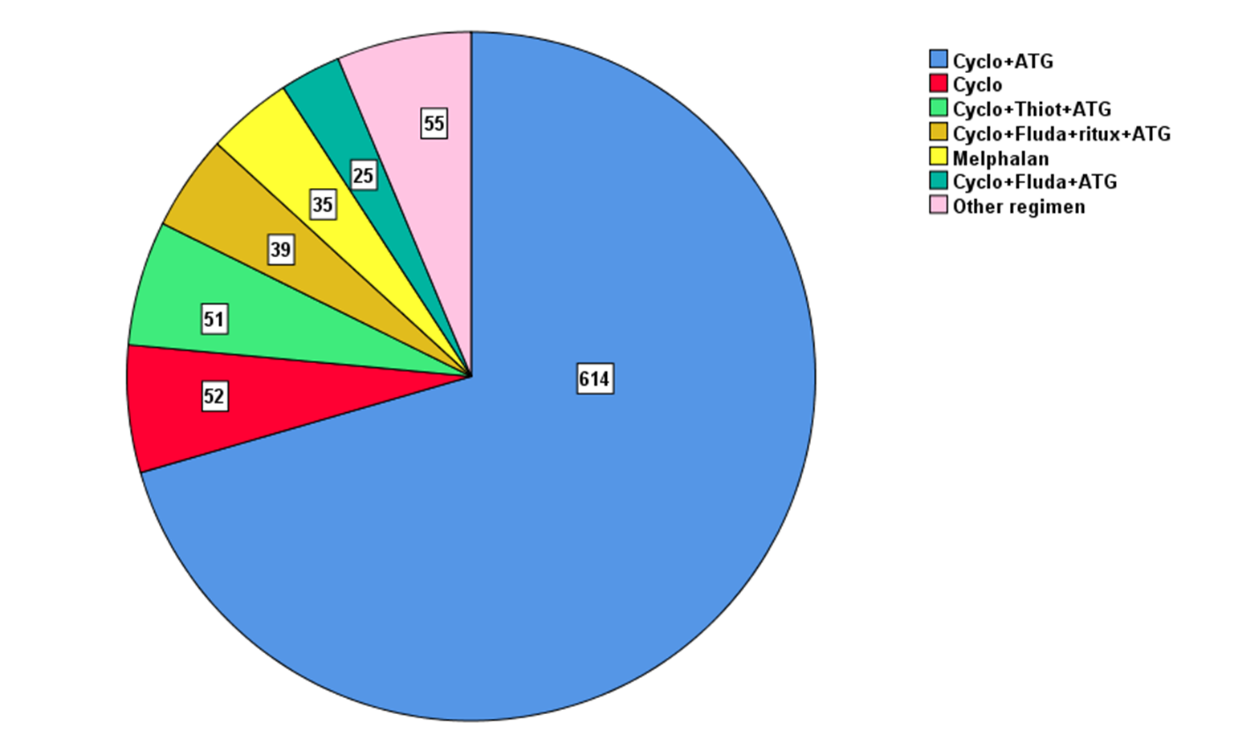


**Supplementary Figure 3:** Conditioning regimens for HCT in rheumatic indications reported to the EBMT registry between 2004 and 07/2023. ATG, anti-thymocyte globulin; Cyclo, cyclophosphamide; Fluda, fludarabine; Thiot, thiotepa, Ritux, rituximab.


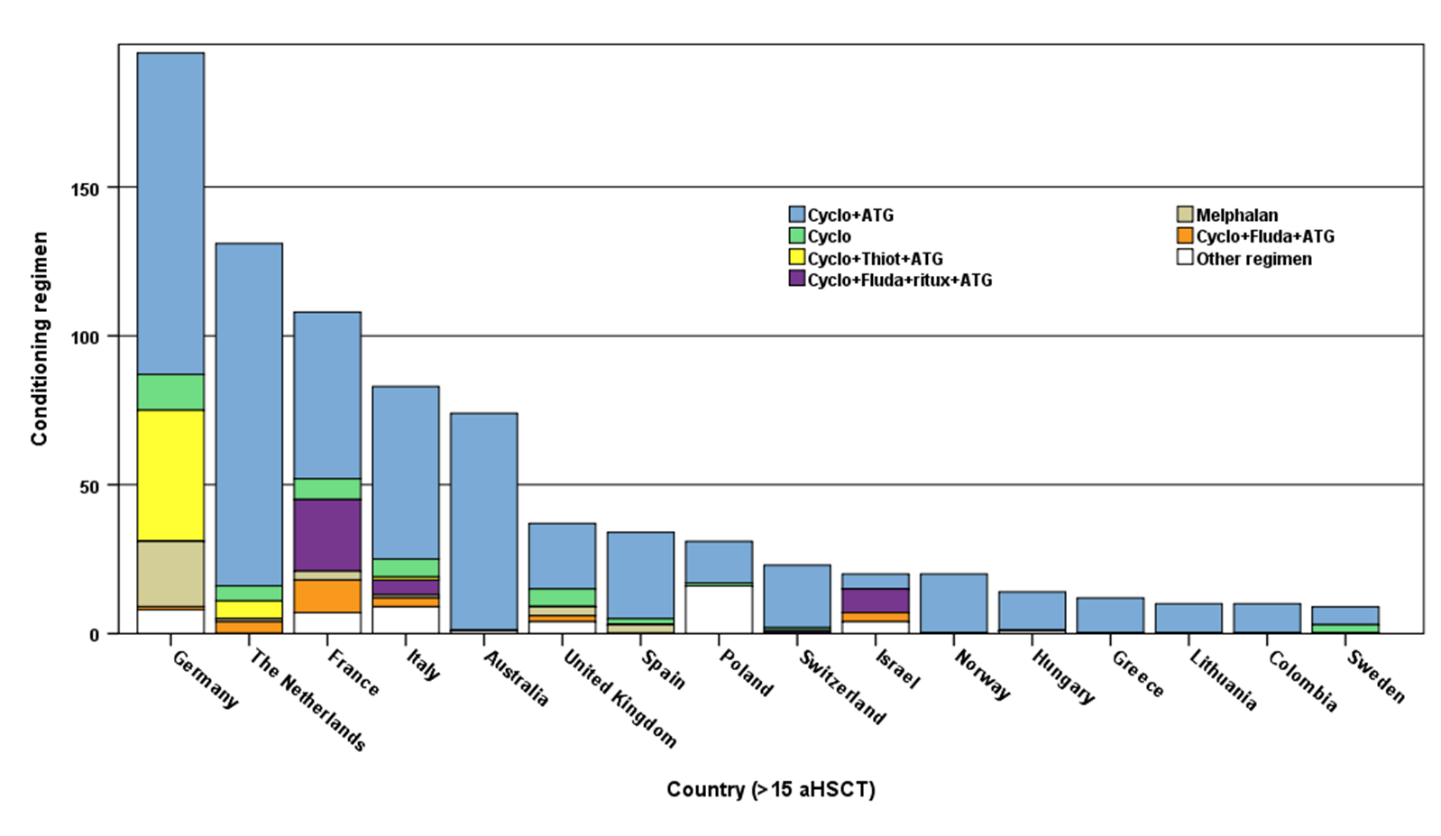


**Supplementary Figure 4:** Distribution of conditioning regimens applied for RMD according to country reported to the EBMT registry between 2004 and 07/2023.


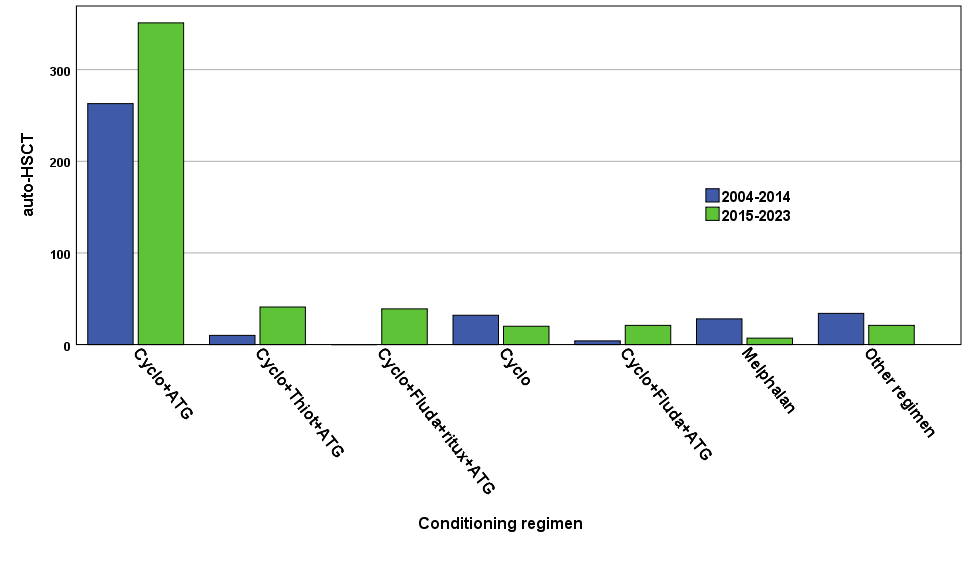


**Supplementary Figure 5:** Number of conditioning regimens applied for RMDs between 2004-2014 (n=371) vs. 2015-07/2023 (n=500) reported to the EBMT registry.


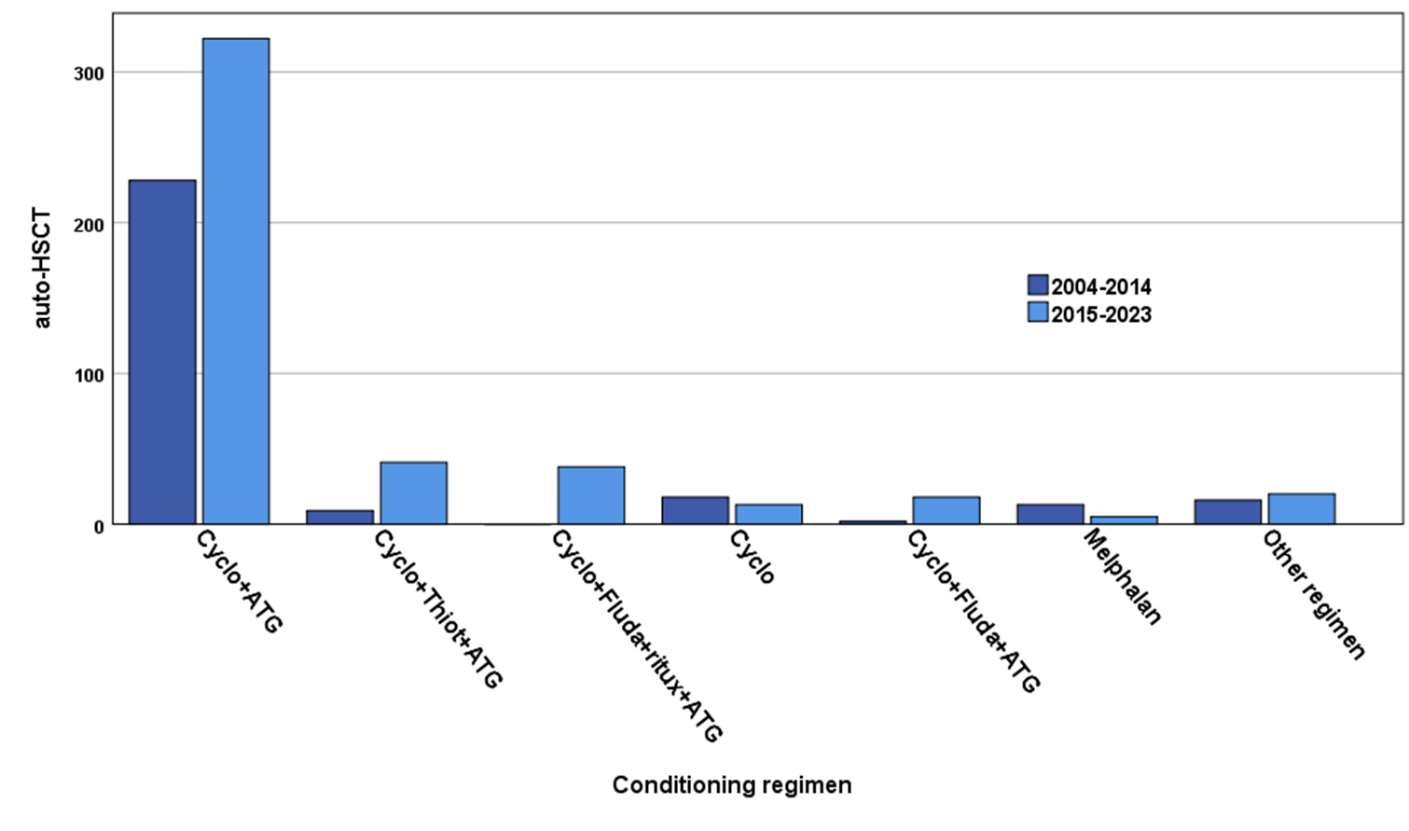


**Supplementary Figure 6:** Number of conditioning regimens applied for systemic sclerosis between 2004-2014 (n=286) vs. 2015-07/2023 (n=457) reported to the EBMT registry.


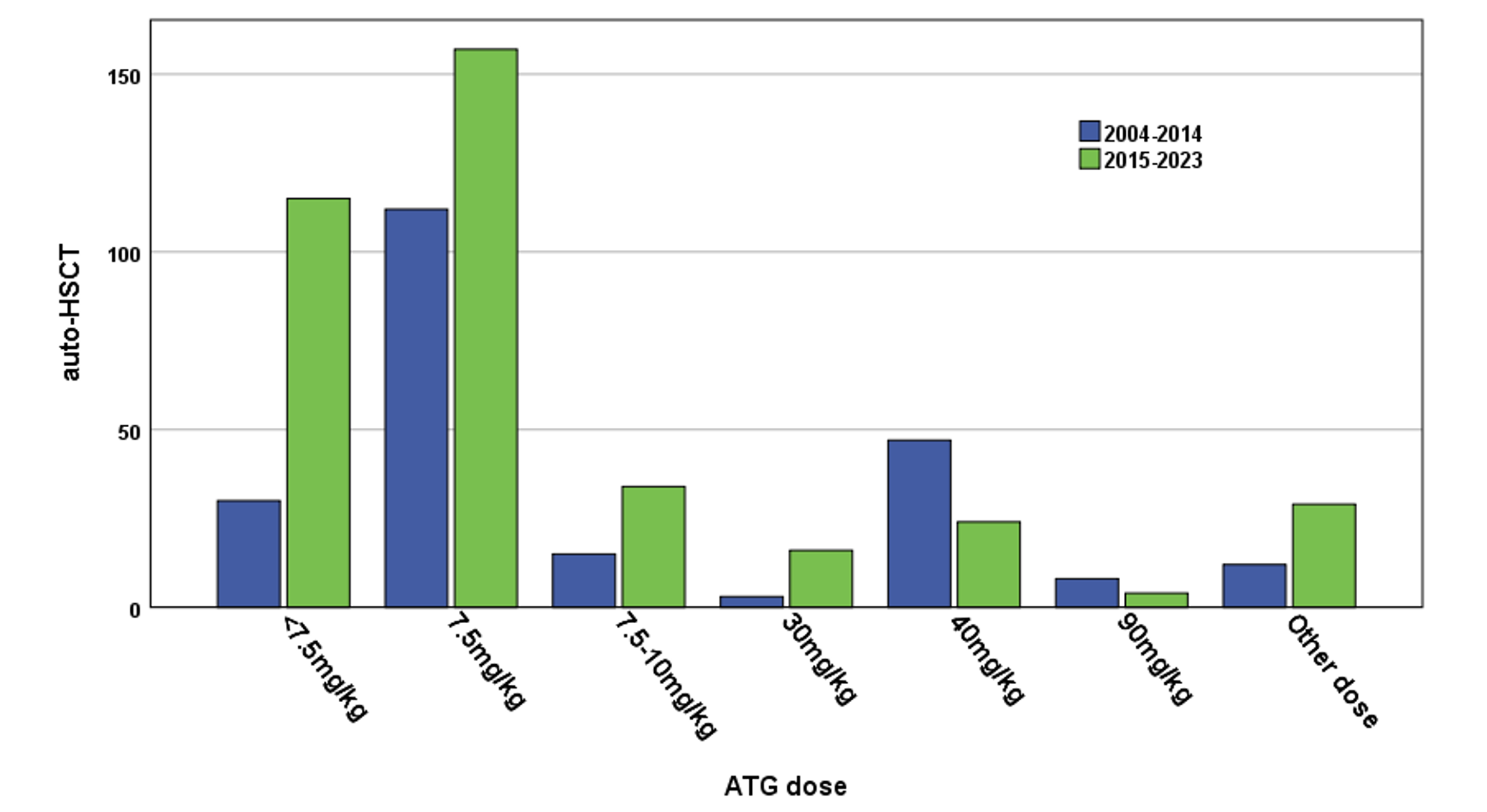


**Supplementary Figure 7:** Doses of anti-thymocyte globulin (ATG) applied for RMD during conditioning 2004-2014 vs. 2015-07/2023 (missing data n=159). Doses until 10mg/kg represent ATG Thymoglobuline®, doses 30-90mg/kg ATG Grafalon®.


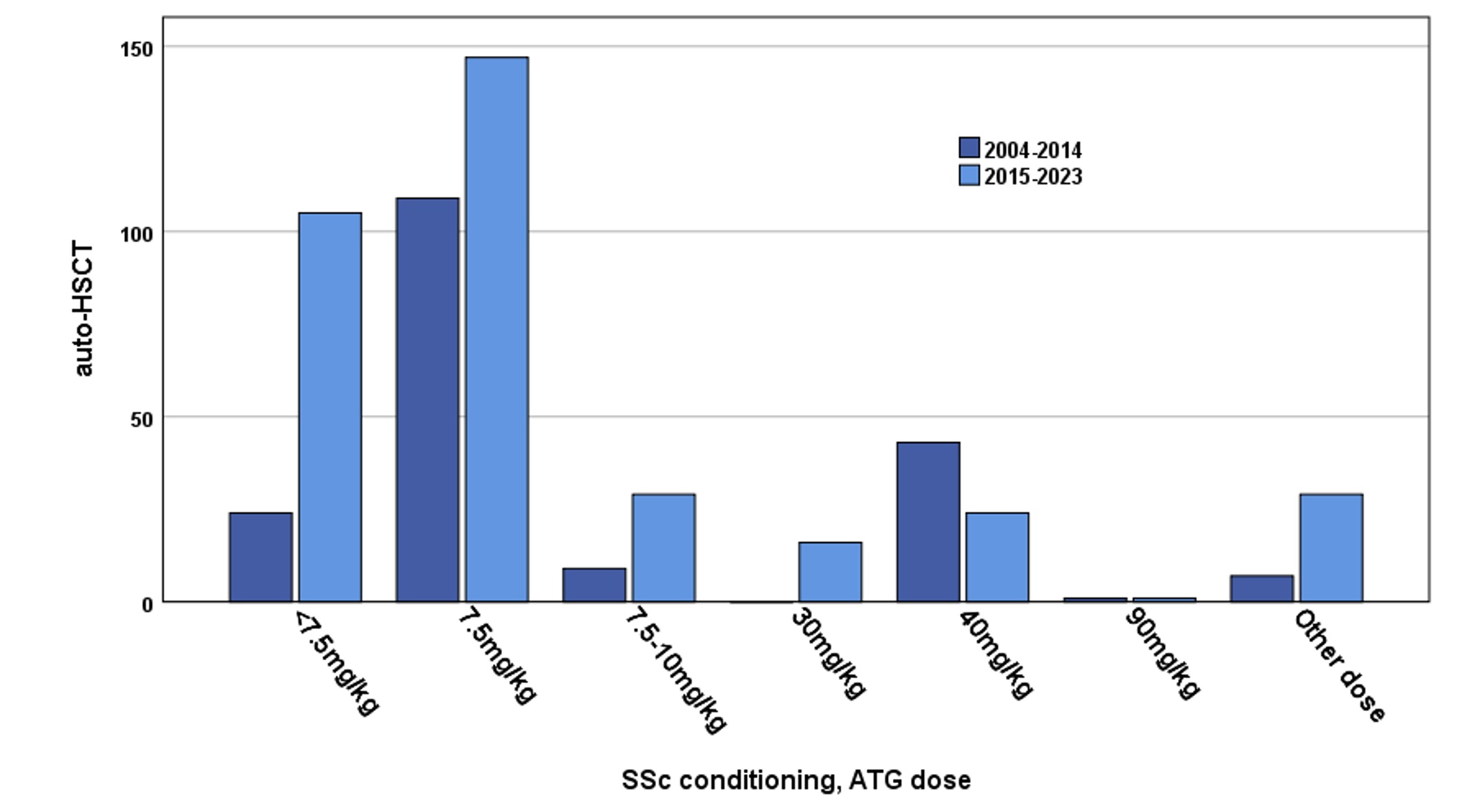


**Supplementary Figure 8:** Doses of anti-thymocyte globulin (ATG) applied for RMD during conditioning 2004-2014 vs. 2015-07/2023 (missing data n=141). Doses until 10mg/kg represent ATG Thymoglobuline®, doses 30-90mg/kg ATG Grafalon®.


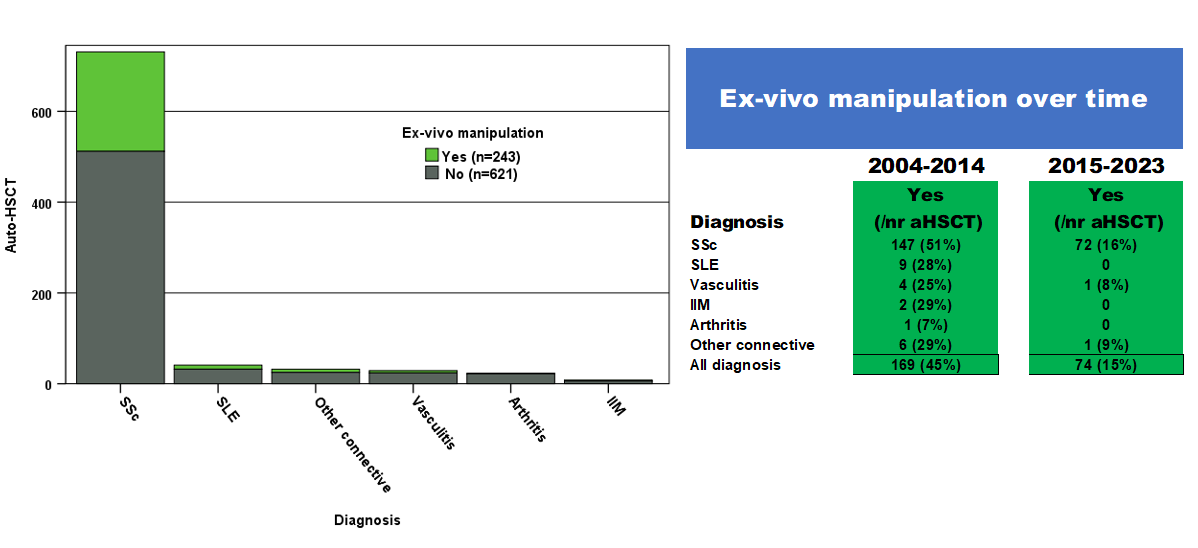


**Supplementary Figure 9:** Number of transplants using ex vivo manipulation (positive selection of CD34+ haematopoietic stem cells) or no ex vivo manipulation according to indication (left graphic, missing data n=41) or time (right table).

**Supplementary Table 1**: **Considerations for HSCT in Idiopathic inflammatory myopathy (IIM) and inflammatory arthritis**

| **Idiopathic inflammatory myopathy** | | | |
| --- | --- | --- | --- |
| *•* Diagnosis of IIM according to 2017 EULAR/ACR classification criteria^1^ with either  - active myositis according to elevated CK levels, MRI or biopsy, with or without the presence of interstitial lung disease OR  - in case of amyopathic disease, presence of ILD is mandatory  *•* Failure of treatment with gluco-corticoids and at least 2 of the following treatments for at least 6 months: IVIG, mycophenolate mofetil, methotrexate, azathioprine, cyclophosphamide, calcineurin inhibitors, or rituximab | • General contraindications (Table 2)  • Life-threatening end-organ damage defined as:  - FVC <45% and/or DLCO (corrected for Hb) < 40% predicted  - LVEF <40% cardiac echocardiography or uncontrolled arrythmias | • Consider risk of concomitant malignancy  • Caution is required for patients with cardiomyopathy | • MMT-8 score^2^ for muscle strengths  • Lung function and CT scan in pulmonary involvement  • Echocardiography and cardiac MRI in cardiomyopathy  • Laboratory assessment of CK, CrP  • Serology: disease-specific autoantibodies, such as Anti-Mi-2, SRP, MDA-5, Jo-1 (among others) |
| **Inflammatory arthritis** | | | |
| *•* Diagnosis of rheumatoid arthritis with systemic manifestations, such as Felty syndrome, or AOSD with life- or organ-threatening involvement and a failure of treatment with glucocorticoids and at least two lines of standard immunosuppressive or immunomodulatory treatment | *•* Life-threatening end-organ damage according to recommendations in the general section | *•* Caution is required for patients with fever and neutropenia.  *•* Consider HLH complicating AOSD | *•* CDAI and DAS-28 score for activity assessment  *•* X-ray, MRI and/or ultrasound to visualize joint damage  *•* Autoantibody levels, e.g. ACPA, rheumatoid factor, inflammation markers, e.g. CrP, ESR, Ferritin, WBC |

Abbreviations: ACPA, anti-citrullinated peptide antibodies; ACR, American College of Rheumatology; AOSD, adult-onset Still's disease; CDAI, clinical disease activity index; CK, creatine kinase; CrP, C-reactive protein; CT, computed tomography; DAS-28, Disease Activity Score 28 ; DLCO, diffusing capacity for carbon monoxide; ESR, erythrocyte sedimentation rate; EULAR, European Alliance of Associations for Rheumatology; FVC, forced vital capacity; HLH, Hemophagocytic lymphohistiocytosis; ILD, interstitial lung disease; IVIG, intravenous immunoglobulin; MMT-8, Muscular Memory Test 8; MRI, Magnetic resonance imaging; LVEF, left ventricular ejection fraction; WBC, white blood cell count.

# **Supplementary Table 2: Studies for HSCT in systemic sclerosis (SSc).**

# Summary of literature on phase 1/2 to phase 3 studies and multicentre registry analysis in the time from 2004 and 2024. Search with keywords: systemic sclerosis; HSCT; stem cell transplantation; stem-cell transplantation

# Papers found: 136. Papers after selection: 27.

| **Author (year)** | **Inclusion criteria** | **Study Design** | **Disease duration at HSCT** | **CD34 selection** | **Patient number** | **Control group** | **Outcome measures** | **Transplant-related mortality** | **Non-transplant-related deaths and leading causes of death** | **Underlined risk factors for mortality** | **Study endpoint** |
| --- | --- | --- | --- | --- | --- | --- | --- | --- | --- | --- | --- |
| Pyka (2024)^3^ | Progressive systemic sclerosis with severe organ involvement | Retrospective study | <12 months | Not specified | 32 | None | OS, TRM, response rates | 6 deaths (18.8%) related to HD-ASCT | Not specified | Renal impairment, age ≥ 55, high comorbidity index | OS, TRM, PFS, risk factors for mortality |
| Keret (2023)^4^ | Early diffuse systemic sclerosis with lung fibrosis, skin thickening (mRSS), and major organ involvement (lungs, kidneys, heart) | Retrospective comparison of AHSCT vs combination therapy (Rituximab + MMF) | <5 years for most patients | Not specified | 16 | 21 patients treated with rituximab and MMF | mRSS reduction (>25%), FVC improvement (>10%) and EFS at 24 months | Three transplant-related deaths (18.7%) | None in combination group; deaths from septic shock, renal crisis, and cardiogenic shock in HSCT group | Major organ involvement (heart, lungs, kidneys) | EFS, mRSS and FVC improvement at 24 months |
| Pecher (2023)^5^ | Patients with systemic sclerosis undergoing mobilization for HSCT | Retrospective single-centre study | Median 3 years | Yes | 32 | None | CD34^+^ stem cell count, mobilization efficiency, adverse events | 1 death during mobilization | None | Low body weight, prior rituximab, cardiac involvement | Stem cell mobilization efficiency, safety outcomes |
| Santana-Gonçalves (2022)^6^ | Diffuse systemic sclerosis with progressive disease | Retrospective observational study | Median 2 years | Not specified | 27 | None | mRSS, FVC, DLCO, serum and skin biomarkers (e.g. E-selectin, VEGFA, IL-6) | 1 death due to transplant-related complications | None | Severe pulmonary involvement, diffuse skin involvement | Improvement in mRSS, skin and vascular biomarkers |
| Zanin-Silva (2022)^7^ | Diffuse systemic sclerosis with skin and/or lung involvement | Retrospective observational study with tissue remodelling analysis | Median 2 years | No | 39 | None | Skin fibrosis, collagen density, mRSS, serum markers (MMPs, TIMP-1, PDGF-AA) | 1 death from transplant-related complications | None | Severe lung involvement | Improvement in skin thickness, collagen density, mRSS, remodelling markers |
| Ciaffi (2022)^8^ | Systemic sclerosis-associated interstitial lung disease (SSc-ILD) | Retrospective observational study | Median 2.5 years | Not specified | 20 | 31 patients treated with cyclophosphamide | HRCT lung changes, pulmonary function tests (PFTs), FVC, DLCO | 3 deaths related to treatment complications (sepsis, multiple organ failure) | 1 death due to disease progression | Severe lung involvement, diffuse cutaneous SSc, prior immunosuppressive treatment | HRCT improvement, PFT stabilization |
| Henrique-Neto (2021)^9^ | Severe systemic sclerosis with skin and/or interstitial lung disease despite immunosuppressive therapy | Longitudinal retrospective study | Median 2 years (range 1-7) | Not specified | 70 | No control group | mRSS, FVC, DLCO, survival | 3 transplant-related deaths (due to sepsis, cyclophosphamide-induced cardiotoxicity, and cholecystitis) | 9 additional deaths due to disease reactivation | Severe baseline disease, cardiac involvement | Improvement in mRSS and FVC, progression-free survival |
| Henes (2021)^10^ | Systemic sclerosis with organ involvement, rapidly progressing disease | Prospective, multicentre non-interventional trial | Median 24 months | Yes, in 35 (43.8%) patients | 88 | No control group | PFS, NRM | NRM of 6.25%, 4  out of 5 deaths related to early cardiac  event | None | Severe organ involvement (lungs, heart) | PFS of 81.8% and OS of 90% at 2 years |
| Costa Pereira (2020)^11^ | Patients with severe systemic sclerosis with pulmonary and/or skin involvement | Prospective observational study | Median 34 months | No | 27 | No control group | Skin score (mRSS), mouth opening, hand grip strength, range of motion, quality of life | None reported | None | None | Significant improvement of mRSS, mouth opening hand function and quality of life |
| Burt (2021)^12^ | Diffuse systemic sclerosis with poor cardiac function, right heart catheterization PASP > 40 mmHg or mPAP > 25 mmHg at rest | Prospective, single-centre study, non-randomized, open-label | Mean 70.5 months (range 11–204) | No | 42 | No control group | Survival, mRSS, FVC, TLC, DLCO, relapse rates | 1 death (2.4%) during transplant due to myocardial infarction | No post-discharge relapse deaths in rituximab group; 2 relapse deaths in non-rituximab group | Cardiac involvement, myocardial fibrosis, high mPAP | 1-year survival, skin score improvement (mRSS), lung function improvement (FVC, TLC) |
| Van Bijnen (2020)^13^ | Severe systemic sclerosis with poor prognosis | Long-term multicentre, retrospective study | Median 1.5 years since first non-Raynaud symptom | Yes | 92 | ASTIS trial comparison with cyclophosphamide-treated patients | EFS, mortality, organ failure | 11% (10/92), primarily cardiorespiratory failure | 7 deaths from disease progression and other causes | Male sex, older age, LVEF <50%, HCT-CI score | EFS at 5, 10, 15 years, skin and pulmonary function improvement |
| Assassi (2019)^14^ | Severe diffuse systemic sclerosis with lung or skin involvement | Randomized controlled trial (SCOT study) | ≤5 years | Yes | 62 | Cyclophosphamide 12-month regimen | Gene expression, molecular signatures, FVC, mRSS | 3% mortality post-transplant | None specified | Pulmonary involvement, severe disease | Gene expression normalization, improvement in mRSS and FVC |
| Ayano (2019)^15^ | Severe systemic sclerosis, moderate-to-severe skin sclerosis, with pulmonary, cardiac, or renal involvement | Post hoc analysis of a phase I/II clinical trial | Median 1.4 years (range 1.2-2.8) | Yes in 11 (58%) patients | 19 | No control group | Improvement of mRSS, pulmonary function (FVC and DLCO) and survival rates | No treatment-related deaths | 2 deaths due to progression of interstitial pneumonia, bacterial pneumonia | Interstitial pneumonia, bacterial infections | Improvement in mRSS, FVC, PFS, OS |
| Helbig (2018)^16^ | Systemic sclerosis with poor prognosis, organ involvement | Observational study | Median 14 months (range 2–85) | Yes | 18 | No control group | mRSS, FVC, overall survival, toxicity and safety profiles | 4 deaths early after transplant (due to pneumonia, myocardial infarction) | 3 deaths due to disease progression and heart attack | Cardiac involvement, infectious complications | Reduction in mRSS, stable lung function, survival rates |
| Nakamura (2018)^17^ | Diffuse systemic sclerosis, disease duration <3 years, mRSS ≥ 15, or refractory digital ulcer/ILD | Phase 2 clinical trial with long-term follow-up | Median 22.5 months (range 8-36) | Yes, in 5 (36%) patients | 14 | No control group | mRSS reduction (>50% within 6 months), event-free survival, pulmonary function | 1 death due to cardiomyopathy | None reported | Cardiac involvement, anti-RNA polymerase III antibodies | EFS, OS, reduction in mRSS, stabilization of ILD |
| Arruda (2018)^18^ | Severe systemic sclerosis (SSc) with major organ involvement | Prospective study with immune monitoring | Median 2.2 years | No | 31 | None | Immune reconstitution | None reported | None reported | None specified | Immune reconstitution, improvement in TCR diversity and Breg numbers |
| Sullivan (2018)^19^ | Severe diffuse systemic sclerosis with pulmonary or renal involvement | Randomized phase 2 trial (HSCT vs. cyclophosphamide) | ≤5 years | Yes | 36 | 39, cyclophosphamide i.v. 12-months regimen | Global rank composite score, EFS, FVC, mRSS, HAQ-DI score | 3% at 54 months, 6% at 72 months | None reported | Pulmonary and renal involvement, lower FVC/DLCO, severe disease | Global rank composite score, event-free survival, overall survival at 72 months |
| Del Papa (2017)^20^ | Rapidly progressive diffuse cutaneous systemic sclerosis with poor response to immune-suppressive therapies | Retrospective cohort analysis | Median 24 months (range 10-48) | Yes | 18 | 36 control patients treated with cyclophosphamide or other immunosuppressive therapies | Skin fibrosis (mRSS), DLCO, ESSG scoring system, OS | 1 transplant-related death (5.6%) | 1 death after 34 months due to cardiac arrhythmia | Severe heart involvement, reduced DLCO | mRSS improvement, stable DLCO, prolonged survival in AHSCT group vs control |
| Michel (2016)^21^ | Severe diffuse systemic sclerosis with skin and lung involvement | Longitudinal observational study | Mean 4 years | Yes | 20 | None | mRSS, serum cytokines (IL-6, IL-8, VEGF, TGF-β1) | None reported | None reported | None specified | Reduction in mRSS, improvement in cytokine profiles, lung function stabilization |
| Van Laar (2014)^22^ | Diffuse cutaneous sclerosis, disease duration max. 4 years and mRSS ≥ 15 and and  Involvement of heart, lung or kidney | Prospective, randomized phase 3 study | Mean 1.4 years | Yes | 79 | 77 patients, treated with 750mg/m^2^ i.v. cyclophosphamide for 12 months | EFS, improvement of mRSS, lung function and quality of life (HAQ-DI) | Eight deaths (10.1% of ITT population) | 9 deaths due to disease progression,  1 cerebro-vascular disease, 1 malignancy | Cardiac involvement, PAH | Superiority of HSCT in EFS, OS, and improvement of mRSS, lung function and quality of life compared to control group |
| Henes (2014)^23^ | Systemic sclerosis with severe cardiac involvement, presence of myocardial fibrosis, pathological ECG, and elevated troponin values | Single-centre, prospective | Median 1.8 years (range 0.5–4.5) | Yes | 6 | None | Reduction in mRSS, pulmonary function improvement (TLC), overall survival | None observed within the 100 days post-transplant | One death after 1.6 years due to disease progression (PAH and skin thickening) | Cardiac involvement, pulmonary arterial hypertension | Reduction in mRSS, improvement in pulmonary function, PFS and OS |
| Moore (2012)^24^ | Severe systemic sclerosis with rapid progression despite prior cyclophosphamide therapy | Retrospective observational study | Median 16 months (range 10-35) | Yes | 10 | None | mRSS, HAQ, VAS, lung function | None reported at 1 year | 2 patients died of progressive disease | None specified | Significant reduction in mRSS, VAS, HAQ; stable lung function |
| Burt (2011)^25^ | Patients until the age of 60 years with diffuse systemic sclerosis and internal organ involvement or skin involvement with pulmonary involvement. | Open-label, randomised phase 2 trial | Median 13.6 months (range 2–33) | No | 10 | 9 patients, treated with 1.0 g/m² i.v. cyclophosphamide monthly for 6 months | Decrease in mRSS (>25%) or increase in FVC (>10%) at 12 months | No deaths reported during follow-up | No deaths occurred in either group | No mortality observed; early intervention before cardiac dysfunction recommended | Improvement in mRSS (>25%) or FVC (>10%) at 12 months; sustained improvement for up to 2 years |
| Tsukamoto (2011)^26^ | Severe diffuse systemic sclerosis with internal organ involvement | Prospective, single-centre study | <4 years | Yes | 11 | None | mRSS, serum anti-Scl-70, Th1/Th2 balance, lung function | None reported | 1 death due to progression of interstitial pneumonia | Advanced interstitial pneumonia | Th1/Th2 ratio, improvement in mRSS and lung function |
| Vonk (2008)^27^ | Severe diffuse systemic sclerosis with major organ involvement and poor prognosis | Long-term follow-up study | Median 2 years (range 0.8–13) | Yes | 26 | None, follow-up study | mRSS, pulmonary function (DLCO, VC), WHO performance status | 2 deaths (7.1%) within 6 months | 3 deaths from disease progression and other causes (lung cancer, relapse) | Severe organ involvement, disease progression | EFS at 5 and 7 years, mRSS, WHO performance status, lung function |
| Farge (2005)^28^ | Severe systemic sclerosis with early visceral involvement | Phase 1/2 clinical trial | Mean 3.5 years | Yes | 7 | None | Immune reconstitution, mRSS, lung function | None reported | None reported | None specified | Immune reconstitution, improvement in mRSS and organ function |
| Farge (2004)^29^ | Severe systemic sclerosis with early visceral involvement (lung, kidney, heart) or life-threatening limited systemic sclerosis with pulmonary fibrosis | Prospective, multicentre study from EBMT/EULAR registry | Median 36 months (2.2–159.4) | In 87% of patients | 57 | None | Skin score, pulmonary function (VC, TLCO), overall survival, disease progression | 5 deaths 8.7%) | 8 deaths related to disease progression | Low LVEF, pulmonary artery pressure > 30 mmHg | Skin score improvement, progression-free survival, overall survival, disease progression probability |

# Abbreviations: Breg, regulatory B cells; EFS, event-free survival; ESSG, European Scleroderma Study Group; EULAR, European Alliance of Associations for Rheumatology; FVC, forced vital capacity; HAQ, Health Assessment Questionnaire; HCT-CI, Haematopoietic Cell Transplantation-specific Comorbidity Index; LVEF, left ventricular ejection fraction; mRSS, modified Rodnan skin score; NRM, non-relapse mortality; OS, overall survival; PAH, pulmonary arterial hypertension; PFS, progression-free survival; TLC, total lung capacity; TLCO, carbon monoxide transfer factor; TRM, transplant-related mortality; VAS, visual analog scale; VC, vital capacity; WHO world health organisation.

# **Supplementary Table 3: Studies for HSCT in systemic lupus erythematosus (SLE).**

# Summary of literature on phase 1/2 to phase 3 studies and multicentre registry analysis in the time from 2004 and 2024.

# Search with keywords: SLE; lupus; haematopoietic stem cell transplantation

# Papers found: 126. Papers after selection: 11.

| Author (year) | Inclusion criteria | Study Design | Patients  (number) | Conditioning regimen | HSC dose | CD34 selection | Follow-up  (months) | AE after HCT | Endpoints |
| --- | --- | --- | --- | --- | --- | --- | --- | --- | --- |
| Goklemez (2022)^30^ | Refractory disease, for LN failure of Cy or MMF for 6 months | Single-centre, prospective | 8 | RTX (750mg/m2), Cy (1200 mg/m^2^), Flu 30mg/m^2^) | 4.9 x 10^6^/kg | yes | 1.4 | TRM 25% (2 death from infection and MOF) | 5/8 patients with CR at 2 years which persisted and 1/8 patient with partial response and later relapse |
| Huang (2019)^31^ | Active lupus nephritis with failure of Cy, MMF or CsA for 6 months | Single-centre, prospective | 22 | Cy (160 mg/kg) + rATG (7.5 mg/kg) | 7.3 x 10^6^/kg | No | 5 | TRM 5% | 82% CR and 5% PR for lupus nephritis, PFS of 53% and relapse incidence of 27% at 5 years |
| Burt (2018)^32^ | Active disease despite Cy or MMF for 6 months | Single-centre, prospective | 30 | Cy (200 mg/kg) + ALE (60 mg/kg) 4/30 pts. and Cy (200 mg/kg) + ATG (5.5 mg/kg), + RTX 1g 26/30 pts. | NA | No | 5 | TRM 0% | PFS 0% in CYC/ALE and 62% in CYC/ATG/RTX group at 5 years post-HSCT |
| Cao (2017)^33^ | Failed therapy, including Cy for 3 months | Single-centre, prospective | 22 | Cy (100–200 mg/kg) + ATG (2–10 mg/kg) | 7.6 x 10^6^/kg | yes | 9.4 | TRM 0% | Progression-free survival of 68% at 5 years |
| Leng (2017)^34^ | Active disease | Single-centre, prospective | 24 | Cy (200mg/kg) + ATG (90mg/kg) or Cy + TBI (4-6Gy) | > 2.0 x 10^6^/kg | yes | 10 | TRM 4% | Progression-free survival of 86% at 10 years |
| Alchi (2013)^35^ | Various | Multicentre, retrospective | 28 | Various, Cy/ATG based | 4.2 x 10^6^/kg | yes | 3.3 | 5 deaths within 2 years | Disease-free survival 29%, relapse incidence 56% |
| Alexander (2009)^36^ | Active disease despite 2 DMARDs incl. Cy for 6 months | Single-centre, prospective | 7 | Cy (200 mg/kg), ATG (90 mg/kg) | 2.4 x 10^6^/kg | yes | 5 | 1 Death (aspergillosis) | Disease-free survival 71% at 5 years |
| Gualandi (2007)^37^ | Active SLE | Single-centre, prospective | 7 | Thiotepa, Cy | NA | xx | 10 | TRM 0% | Complete remission 50% |
| Burt (2006)^38^ | Active disease for LN with failure of 6mo Cy and non-renal of 3mo Cy | Single-centre, prospective | 50 | Cy (200 mg/kg), ATG (90 mg/kg) | 5.5 x 106/kg | no | 5 | TRM 2% | Disease-free survival 50% at 5 years |
| Leng (2005)^39^ | Active SLE | Single-centre, prospective | 17 | Cy 200 mg/kg | NA | yes | 4 | TRM 6% | Disease-free survival of 82% at 50 months |
| Jayne (2004)^40^ | Various | Multicentre, retrospective | 53 | Various, CY based | NA | various | 5 | TRM 12% | Remission rate of 55% after 5 years |

Abbreviations: ALE, alemtuzumab; ATG, anti-thymocyte globulin; CsA, cyclosporine A; Cy, cyclophosphamide; Flu, fludarabine; LN, lupus nephritis; MMF, mycophenolate mofetil; RTX, rituximab; TRM, transplant-related mortality.

# **Supplementary Table 4: Studies for HSCT in idiopathic inflammatory myopathy (IIM).**

# Summary of literature on phase 1/2 to phase 3 studies and case reports in the time from 2004 and 2024.

# Search with keywords: myositis; haematopoietic stem cell transplantation.

# Papers found: 62. Papers after selection: 4.

| Author (year) | Inclusion criteria | Study Design | Patients  (number) | Conditioning regimen | HSC dose | CD34 selection | Follow-up  (months) | AE after HSCT | Endpoints |
| --- | --- | --- | --- | --- | --- | --- | --- | --- | --- |
| Storek  (2013)^41^ | Dermatomyositis | Case report | 1 | Cy/ATG | NA | NA | NA | no | No response |
| Henes  (2009)^42^ | Anti-signal recognition particle-positive polymyositis | Case report | 1 | Cy 200 mg/kg + ATG 40mg/kg) | 7.1 x10^6^/kg | yes | 36 | yes | Remission at 3 years |
| Tsukamoto (2006)^43^ | DM, refractory to conventional immunosuppressive treatment | Prospective, open-label phase I/II trial | 1 | Cy 200 mg/kg | 4.9x10^6^/kg | yes | 1 | Sepsis, CMV viraemia | Improvement of lung function at 12mo |
| Oryoji  (2005)^44^ | Rapidly progressive and refractory DM with pulmonary involvement | Case report | 1 | Cy | NA | NA | 1.5 | None | PFS at 18 months |

Abbreviations: ATG, antithymocyte globuline; CMV, cytomegalovirus; Cy, cyclophosphamide; DM, dermatomyositis; PFS, progression-free survival

# **Supplementary Table 5: Studies for HSCT in vasculitis.**

# Summary of literature on phase 1/2 to phase 3 studies and case reports in the time from 2004 and 2024.

# Search with keywords: vasculitis; haematopoietic stem cell transplantation.

# Papers found: 184. Papers after selection: 6.

| Author (year) | Inclusion criteria | Study Design | Patients  (number) | Conditioning regimen | HSC dose | CD34 selection | Follow-up  (months) | AE after HSCT | Endpoints |
| --- | --- | --- | --- | --- | --- | --- | --- | --- | --- |
| Laurent (2022)^45^ | Takayasu arteritis | Retrospective, multicentre | 6 | Cy/ATG | 3.9x10^6^/kg | In 2/6 pts | 9.9 | Ulcerative colitis, | 100% remission at 6 months, which persisted at 12 months in five cases, 4 relapses at a median 27 months |
| Puyade (2021)^46^ | Behçet's disease | Retrospective, multicentre | 9 | Melphalan in 5pts, BEAM in 3 pts, Cy/ATG in 1 patient | Na | Various | 4 | FUO, Pneumonia | CR in 80%, PR remission in 10% and lack of response in 10%, relapse rate 30% |
| Alexander (2020)^47^ | ANCA vasculitis, refractory to standard immunosuppressive treatment including Cy | Retrospective, multicentre | 7 | Cy 200mg/kg + ATG in 5 pts | 5.4x10^6^/kg | In 4/7 pts | 10 | 2 deaths (pulmonary toxicity and sepsis) | OS 71%, PFS of 29% and an RI of 60% at 10 years |
| Statkute (2008)^48^ | GPA, Behçet's disease and neurovascular Sjogren’s syndrome, refractory to Cy for 6months | Single-centre, prospective | 4 | Cy 200 mg/kg + ATG 85.5mg/kg) | 6.9x10^6^/kg | yes | 2 | CMV reactivation, neutropenic fever | 3 pts (GPA, BD and SS) in remission at 24 months |
| Daikeler (2007)^49^ | Various refractory vasculitis | Retrospective, multicenter | 7 | Varios, Cy/ATG based | 3.9x10^6^/kg | In 2/6 pts | 1.8 | CMV and EBV reactivation, DIC, neutropenic fever | 46% complete responses and 46% partial responses |
| Tsukamoto (2006)^43^ | GPA, refractory to conventional immunosuppressive treatment | Prospective, open-label phase I/II trial | 1 | Cy 200 mg/kg | 5.0x10^6^/kg | yes | 16 | none | Orbital granuloma size markedly decreased |

Abbreviations: ANCA, anti-neutrophil cytoplasmic antibodies; ATG, anti-thymocyte globulin; BD, Behçet's disease; BEAM, (carmustine, etoposide, cytarabine, melphalan); CMV, cytomegalovirus; Cy, cyclophosphamide; CR, complete response; DIC, disseminated intravascular coagulation; EBV, Epstein-Barr Virus; FUO, fever of unknown origin; GPA, granulomatosus with polyangiitis; OS, overall survival; RI, relapse incidence; SS Sjogren’s syndrome.

# **Supplementary Table 5: Studies for HSCT in rheumatoid arthritis.**

# Summary of literature on phase 1/2 to phase 3 studies and multicentre registry analysis in the time from 2004 and 2024.

# Search with arthritis; haematopoietic stem cell transplantation.

# Papers found: 44. Papers after selection: 2.

#

| Author (year) | Inclusion criteria | Study Design | Patients  (number) | Conditioning regimen | | HSC dose | CD34 selection | Follow-up  (months) | AE after HSCT | Endpoints |
| --- | --- | --- | --- | --- | --- | --- | --- | --- | --- | --- |
| Verburg (2005)^50^ | RA with failure to respond to >4 antirheumatic drugs, incl. maximal tolerable dose of methotrexate | Single-centre, prospective | 7 | Cy 200mg/kg | NA | | yes | 12 | None | 5/7 patients attaining a good response based on the EULAR response criteria at 3 months but then progressed |
| Snowden (20024)^51^ | Refractory RA | Multicentre, retrospective | 73 | Various, Cy 200mg/kg in 62 pts | NA | | No | 12 | 1 death from infection and NSCLC | 67% achieved at least ACR 50% at 12 months, most pts restarted DMARD within 6 months |

Abbreviations: ACR, American College of Rheumatology; Cy, cyclophosphamide; DMARD, disease-modifying drug; EULAR, European Alliance of Associations for Rheumatology; NSCLC, Non-small cell lung cancer; RA, rheumatoid arthritis.

**Supplementary References:**

1. Lundberg IE, Tjärnlund A, Bottai M, et al. 2017 European League Against Rheumatism/American College of Rheumatology classification criteria for adult and juvenile idiopathic inflammatory myopathies and their major subgroups. *Ann Rheum Dis* 2017; **76**(12): 1955-64.

2. Rider LG, Koziol D, Giannini EH, et al. Validation of manual muscle testing and a subset of eight muscles for adult and juvenile idiopathic inflammatory myopathies. *Arthritis Care Res (Hoboken)* 2010; **62**(4): 465-72.

3. Pyka V, Vangala DB, Mika T, et al. High-dose chemotherapy and autologous hematopoietic stem cell transplantation for progressive systemic sclerosis: a retrospective study of outcome and prognostic factors. *J Cancer Res Clin Oncol* 2024; **150**(6): 301.

4. Keret S, Henig I, Zuckerman T, et al. Outcomes in progressive systemic sclerosis treated with autologous hematopoietic stem cell transplantation compared with combination therapy. *Rheumatology (Oxford)* 2024; **63**(6): 1534-8.

5. Pecher AC, Ach KR, Vogel W, Henes JC. Mobilization with reduced cyclophosphamide for autologous stem cell transplantation is feasible in patients with systemic sclerosis. *Rheumatology (Oxford)* 2023; **62**(Si): Si107-si13.

6. Santana-Gonçalves M, Zanin-Silva D, Henrique-Neto Á, et al. Autologous hematopoietic stem cell transplantation modifies specific aspects of systemic sclerosis-related microvasculopathy. *Ther Adv Musculoskelet Dis* 2022; **14**: 1759720x221084845.

7. Zanin-Silva DC, Santana-Gonçalves M, Kawashima-Vasconcelos MY, et al. Autologous hematopoietic stem cell transplantation promotes connective tissue remodeling in systemic sclerosis patients. *Arthritis Res Ther* 2022; **24**(1): 95.

8. Ciaffi J, van Leeuwen NM, Boonstra M, et al. Evolution of Systemic Sclerosis-Associated Interstitial Lung Disease One Year After Hematopoietic Stem Cell Transplantation or Cyclophosphamide. *Arthritis Care Res (Hoboken)* 2022; **74**(3): 433-41.

9. Henrique-Neto Á, Vasconcelos MYK, Dias JBE, et al. Hematopoietic stem cell transplantation for systemic sclerosis: Brazilian experience. *Adv Rheumatol* 2021; **61**(1): 9.

10. Henes J, Oliveira MC, Labopin M, et al. Autologous stem cell transplantation for progressive systemic sclerosis: a prospective non-interventional study from the European Society for Blood and Marrow Transplantation Autoimmune Disease Working Party. *Haematologica* 2021; **106**(2): 375-83.

11. Costa-Pereira KR, Guimarães AL, Moraes DA, et al. Hematopoietic Stem Cell Transplantation Improves Functional Outcomes of Systemic Sclerosis Patients. *J Clin Rheumatol* 2020; **26**(7S Suppl 2): S131-s8.

12. Burt RK, Han X, Quigley K, et al. Cardiac safe hematopoietic stem cell transplantation for systemic sclerosis with poor cardiac function: a pilot safety study that decreases neutropenic interval to 5 days. *Bone Marrow Transplant* 2021; **56**(1): 50-9.

13. van Bijnen S, de Vries-Bouwstra J, van den Ende CH, et al. Predictive factors for treatment-related mortality and major adverse events after autologous haematopoietic stem cell transplantation for systemic sclerosis: results of a long-term follow-up multicentre study. *Ann Rheum Dis* 2020; **79**(8): 1084-9.

14. Assassi S, Wang X, Chen G, et al. Myeloablation followed by autologous stem cell transplantation normalises systemic sclerosis molecular signatures. *Ann Rheum Dis* 2019; **78**(10): 1371-8.

15. Ayano M, Tsukamoto H, Mitoma H, et al. CD34-selected versus unmanipulated autologous haematopoietic stem cell transplantation in the treatment of severe systemic sclerosis: a post hoc analysis of a phase I/II clinical trial conducted in Japan. *Arthritis Res Ther* 2019; **21**(1): 30.

16. Helbig G, Widuchowska M, Koclęga A, et al. Safety profile of autologous hematopoietic stem cell mobilization and transplantation in patients with systemic sclerosis. *Clin Rheumatol* 2018; **37**(6): 1709-14.

17. Nakamura H, Odani T, Yasuda S, et al. Autologous haematopoietic stem cell transplantation for Japanese patients with systemic sclerosis: Long-term follow-up on a phase II trial and treatment-related fatal cardiomyopathy. *Mod Rheumatol* 2018; **28**(5): 879-84.

18. Arruda LCM, Malmegrim KCR, Lima-Júnior JR, et al. Immune rebound associates with a favorable clinical response to autologous HSCT in systemic sclerosis patients. *Blood Adv* 2018; **2**(2): 126-41.

19. Sullivan KM, Goldmuntz EA, Keyes-Elstein L, et al. Myeloablative Autologous Stem-Cell Transplantation for Severe Scleroderma. *N Engl J Med* 2018; **378**(1): 35-47.

20. Del Papa N, Onida F, Zaccara E, et al. Autologous hematopoietic stem cell transplantation has better outcomes than conventional therapies in patients with rapidly progressive systemic sclerosis. *Bone Marrow Transplant* 2017; **52**(1): 53-8.

21. Michel L, Farge D, Baraut J, et al. Evolution of serum cytokine profile after hematopoietic stem cell transplantation in systemic sclerosis patients. *Bone Marrow Transplant* 2016; **51**(8): 1146-9.

22. van Laar JM, Farge D, Sont JK, et al. Autologous hematopoietic stem cell transplantation vs intravenous pulse cyclophosphamide in diffuse cutaneous systemic sclerosis: a randomized clinical trial. *Jama* 2014; **311**(24): 2490-8.

23. Henes JC, Koetter I, Horger M, et al. Autologous stem cell transplantation with thiotepa-based conditioning in patients with systemic sclerosis and cardiac manifestations. *Rheumatology (Oxford)* 2014; **53**(5): 919-22.

24. Moore J, Englert H, Furlong T, Poon T, Milliken S, Ma D. Auto-HSCT induces sustained responses in severe systemic sclerosis patients failing pulse cyclophosphamide. *Bone Marrow Transplant* 2012; **47**(11): 1486-7.

25. Burt RK, Shah SJ, Dill K, et al. Autologous non-myeloablative haemopoietic stem-cell transplantation compared with pulse cyclophosphamide once per month for systemic sclerosis (ASSIST): an open-label, randomised phase 2 trial. *Lancet* 2011; **378**(9790): 498-506.

26. Tsukamoto H, Nagafuji K, Horiuchi T, et al. Analysis of immune reconstitution after autologous CD34+ stem/progenitor cell transplantation for systemic sclerosis: predominant reconstitution of Th1 CD4+ T cells. *Rheumatology (Oxford)* 2011; **50**(5): 944-52.

27. Vonk MC, Marjanovic Z, van den Hoogen FH, et al. Long-term follow-up results after autologous haematopoietic stem cell transplantation for severe systemic sclerosis. *Ann Rheum Dis* 2008; **67**(1): 98-104.

28. Farge D, Henegar C, Carmagnat M, et al. Analysis of immune reconstitution after autologous bone marrow transplantation in systemic sclerosis. *Arthritis Rheum* 2005; **52**(5): 1555-63.

29. Farge D, Passweg J, van Laar JM, et al. Autologous stem cell transplantation in the treatment of systemic sclerosis: report from the EBMT/EULAR Registry. *Ann Rheum Dis* 2004; **63**(8): 974-81.

30. Goklemez S, Hasni S, Hakim FT, et al. Long-term follow-up after lymphodepleting autologous haematopoietic cell transplantation for treatment-resistant systemic lupus erythematosus. *Rheumatology (Oxford)* 2022; **61**(8): 3317-28.

31. Huang X, Chen W, Ren G, et al. Autologous Hematopoietic Stem Cell Transplantation for Refractory Lupus Nephritis. *Clin J Am Soc Nephrol* 2019; **14**(5): 719-27.

32. Burt RK, Han X, Gozdziak P, et al. Five year follow-up after autologous peripheral blood hematopoietic stem cell transplantation for refractory, chronic, corticosteroid-dependent systemic lupus erythematosus: effect of conditioning regimen on outcome. *Bone Marrow Transplant* 2018; **53**(6): 692-700.

33. Cao C, Wang M, Sun J, et al. Autologous peripheral blood haematopoietic stem cell transplantation for systemic lupus erythematosus: the observation of long-term outcomes in a Chinese centre. *Clin Exp Rheumatol* 2017; **35**(3): 500-7.

34. Leng XM, Jiang Y, Zhou DB, et al. Good outcome of severe lupus patients with high-dose immunosuppressive therapy and autologous peripheral blood stem cell transplantation: a 10-year follow-up study. *Clin Exp Rheumatol* 2017; **35**(3): 494-9.

35. Alchi B, Jayne D, Labopin M, et al. Autologous haematopoietic stem cell transplantation for systemic lupus erythematosus: data from the European Group for Blood and Marrow Transplantation registry. *Lupus* 2013; **22**(3): 245-53.

36. Alexander T, Thiel A, Rosen O, et al. Depletion of autoreactive immunologic memory followed by autologous hematopoietic stem cell transplantation in patients with refractory SLE induces long-term remission through de novo generation of a juvenile and tolerant immune system. *Blood* 2009; **113**(1): 214-23.

37. Gualandi F, Bruno B, Van Lint MT, et al. Autologous stem cell transplantation for severe autoimmune diseases: a 10-year experience. *Ann N Y Acad Sci* 2007; **1110**: 455-64.

38. Burt RK, Traynor A, Statkute L, et al. Nonmyeloablative hematopoietic stem cell transplantation for systemic lupus erythematosus. *Jama* 2006; **295**(5): 527-35.

39. Leng XM, Zhao Y, Zhou DB, et al. A pilot trial for severe, refractory systemic autoimmune disease with stem cell transplantation. *Chin Med Sci J* 2005; **20**(3): 159-65.

40. Jayne D, Tyndall A. Autologous stem cell transplantation for systemic lupus erythematosus. *Lupus* 2004; **13**(5): 359-65.

41. Storek J, LeClercq SA, Aaron SL. Lack of sustained response of advanced dermatomyositis to autologous haematopoietic cell transplantation. *Scand J Rheumatol* 2013; **42**(5): 421-2.

42. Henes JC, Heinzelmann F, Wacker A, et al. Antisignal recognition particle-positive polymyositis successfully treated with myeloablative autologous stem cell transplantation. *Ann Rheum Dis* 2009; **68**(3): 447-8.

43. Tsukamoto H, Nagafuji K, Horiuchi T, et al. A phase I-II trial of autologous peripheral blood stem cell transplantation in the treatment of refractory autoimmune disease. *Ann Rheum Dis* 2006; **65**(4): 508-14.

44. Oryoji K, Himeji D, Nagafuji K, et al. Successful treatment of rapidly progressive interstitial pneumonia with autologous peripheral blood stem cell transplantation in a patient with dermatomyositis. *Clin Rheumatol* 2005; **24**(6): 637-40.

45. Laurent C, Marjanovic Z, Ricard L, et al. Autologous hematopoietic stem cell transplantation with reduced-intensity conditioning regimens in refractory Takayasu arteritis: a retrospective multicenter case-series from the Autoimmune Diseases Working Party (ADWP) of the European Society for Blood and Marrow Transplantation (EBMT). *Bone Marrow Transplant* 2020; **55**(11): 2109-13.

46. Puyade M, Patel A, Lim YJ, et al. Autologous Hematopoietic Stem Cell Transplantation for Behçet's Disease: A Retrospective Survey of Patients Treated in Europe, on Behalf of the Autoimmune Diseases Working Party of the European Society for Blood and Marrow Transplantation. *Front Immunol* 2021; **12**: 638709.

47. Alexander T, Samuelson C, Daikeler T, et al. Autologous haematopoietic stem cell transplantation (HSCT) for anti-neutrophil cytoplasmic antibody (ANCA)-associated vasculitis: a retrospective survey of patients reported to European Society for Blood and Marrow Transplantation (EBMT) registry. *Bone Marrow Transplant* 2020; **55**(7): 1512-5.

48. Statkute L, Oyama Y, Barr WG, et al. Autologous non-myeloablative haematopoietic stem cell transplantation for refractory systemic vasculitis. *Ann Rheum Dis* 2008; **67**(7): 991-7.

49. Daikeler T, Kötter I, Bocelli Tyndall C, et al. Haematopoietic stem cell transplantation for vasculitis including Behcet's disease and polychondritis: a retrospective analysis of patients recorded in the European Bone Marrow Transplantation and European League Against Rheumatism databases and a review of the literature. *Ann Rheum Dis* 2007; **66**(2): 202-7.

50. Verburg RJ, Flierman R, Sont JK, et al. Outcome of intensive immunosuppression and autologous stem cell transplantation in patients with severe rheumatoid arthritis is associated with the composition of synovial T cell infiltration. *Ann Rheum Dis* 2005; **64**(10): 1397-405.

51. Snowden JA, Passweg J, Moore JJ, et al. Autologous hemopoietic stem cell transplantation in severe rheumatoid arthritis: a report from the EBMT and ABMTR. *J Rheumatol* 2004; **31**(3): 482-8.
